# Electronics-free soft robotic minitablet for on-demand gastric molecular sensing and diagnostics in vivo

**DOI:** 10.1126/sciadv.aea3309

**Published:** 2026-05-08

**Authors:** Chen Wang, Rui Shi, Anatolii Abalymov, Hao Bao, Thomas Ka-Yam LAM, Zihan Wang, Yongfeng Mei, Zongwei Cai, Xiang-zhong Chen, Sarthak Misra, Venkatasubramanian K. Venkiteswaran

**Affiliations:** ^1^ Surgical Robotics Lab, Department of Biomaterials and Biomedical Technology, University Medical Center Groningen, University of Groningen, Groningen 9713 AV, Netherlands.; ^2^ Department of Biomechanical Engineering, University of Twente, Enschede 7522 NB, Netherlands.; ^3^ State Key Laboratory of Environmental and Biological Analysis, Hong Kong Baptist University, Hong Kong SAR 999077, China.; ^4^ International Institute of Intelligent Nanorobots and Nanosystem, College of Intelligent Robotics and, Advanced Manufacturing, Fudan University, Shanghai 200438, China.; ^5^ Shanghai Frontiers Science Research Base of Intelligent Optoelectronics and Perception, Institute of Optoelectronics, Fudan University, Shanghai 200438, China.; ^6^ State Key Laboratory of Surface Physics, Fudan University, Shanghai 200438, China.; ^7^ Zhejiang Key Laboratory of Extreme Environment Functional Materials, Yiwu Research Institute of Fudan University, Yiwu, Zhejiang 322000, China.; ^8^ Eastern Institute of Technology, Ningbo 315200, China.; ^9^ State Key Laboratory of Photovoltaic Science and Technology, Fudan University, Shanghai 200438, China.

## Abstract

Real-time biomarker sensing and molecular sampling in the stomach can transform how gastrointestinal disorders are diagnosed and managed—yet integrating both capabilities into a single ingestible platform remains a formidable challenge. Existing devices often struggle with size constraints, limited functionality, and the mechanical mismatch between soft, biocompatible materials and rigid electronics. Here, we present SeroTab, an electronics-free soft robotic minitablet that combines real-time pH sensing with on-demand gastric fluid sampling in vivo. Inspired by the gliding motion of penguins through viscous environments, SeroTab features a magnetically actuated, curvature-adaptive body that enables autonomous navigation through complex anatomical geometries to specific gastric regions. Upon reaching its target, a shape memory polymer actuator—triggered wirelessly via radio frequency heating—draws gastric fluid into an internal reservoir (up to 35 microliters). A pH-sensitive hydrogel within the chamber, embedded with biocompatible metal disks, enables ultrasound-based pH readout across a physiologically relevant range (pH 2 to 7) and preserves the sample for untargeted metabolomic profiling. In vivo studies in animal models demonstrate SeroTab’s ability to detect pharmacologically induced pH changes (ΔpH = 4, from 2 to 6) and metabolic shifts (42 detected metabolites) following omeprazole administration. By uniting soft robotics, responsive materials, and wireless actuation, SeroTab paves the way for minimally invasive, outpatient-compatible diagnostics that can advance early screening and streamline clinical decision-making.

## Teaser

SeroTab is an ingestible soft robotic tablet that senses gastric pH in real time and samples stomach fluid on demand.

## INTRODUCTION

The global burden of gastrointestinal (GI) diseases remains substantial, with an estimated 7 billion incident cases and 8 million deaths annually, exacerbated by constrained medical resources and delayed patient access to care (*1*, *2*). GI disorders are common in primary health care, accounting for ~10% of consultations (*3*). Timely and effective GI disease diagnosis and patient triage at the primary care level can alleviate hospital burden and improve patient outcomes (*4*). Many countries adopt tiered health care systems in which general practitioners (GPs) serve as gatekeepers, managing patient flow and easing hospital demand (*5*, *6*). However, GPs often face diagnostic uncertainty with GI conditions, partly due to limited access to advanced tools and the high cost of precision diagnostics (e.g., endoscopic systems, x-ray, and computed tomography), leading to diagnostic delays and further straining hospitals. Basic GI diagnostic tools available in primary care [e.g., fecal occult blood tests, abdominal palpation devices, and ultrasound (US) imaging] are predominantly designed for initial GI screening and general physiological monitoring (*7*). Although these tools aid GPs in detecting potential GI abnormalities, they typically fall short in sensitivity, specificity, or biomarker resolution necessary for definitive diagnosis of complex or organ-specific pathologies.

Among GI diagnostic targets, gastric acid serves as a crucial pathophysiological indicator correlated with a spectrum of GI dysfunctions, including gastroesophageal reflux disease (GERD), gastric ulcer, gastroenteritis, and gastric cancer (*8*–*10*). Clinical assessment of gastric acid secretion requires both rapid and precise examination of gastric juice samples obtained from the lesion site, necessitating on-site detection of physicochemical parameters (e.g., pH, temperature, and pressure) for urgent medical care, alongside comprehensive laboratory analysis of biological omics profiles (e.g., proteomics, metabolomics, and the microbiome) to elucidate disease biomarkers and track disease progression (*11*, *12*). The traditional aspiration test for sampling the gastric juice (*13*) requires the placement of a nasogastric tube into the stomach, leading to patient discomfort and contraindications (*14*). Recent advances in personalized medicine have brought forth orally administered, tetherless electronic capsules [e.g., Heidelberg (*15*), PillCam, and VitalSense capsules], offering noninvasive detection of gastric fluid parameters (*16*–*18*). However, the passive movement of these devices, driven solely by digestive tract peristalsis, limits their ability to conduct targeted sampling and precise sensing (*19*). In addition, these electronic sensing devices face challenges in miniaturization, power supply, and biocompatibility.

Emerging magnetic soft robots combine active control and biocompatibility, enabling precise navigation of small-scale agents within the human body for accurate diagnostics, localized treatment, and therapeutic interventions (*20*–*22*). By programming soft materials with specific magnetization profiles, robots are endowed with locomotion and deformation capabilities, allowing them to adapt to in vivo environments and access confined spaces under the control of external magnetic fields (*23*–*26*). In combination with functional materials and external stimuli, the robots can be equipped for clinical treatment (*19*, *27*, *28*). For instance, magnetically actuated robotic capsules/tablets/pills (*29*) have demonstrated potential in performing liquid sampling (*30*, *31*), tissue biopsy (*32*, *33*), drug delivery (*34*), and microbiome collection (*35*–*37*) at targeted sites. Robots have also been developed with integrated sensing capabilities for rapid diagnosis and real-time physiological monitoring. Methods for sensorizing magnetic soft robotic devices are generally classified into two categories: direct and indirect. Direct sensing entails the integration of microelectronic systems (*34*, *38*–*40*), whereas indirect sensing leverages the mechanical deformation of the robot’s structure in synergy with multimaterial configurations (*41*–*47*), assisted by external medical imaging systems (e.g., US, x-ray, and computed tomography) (*48*, *49*). The direct sensing approach encounters challenges related to the integration of soft and rigid materials as well as electromagnetic interference, whereas the latter demands innovations in materials and communication strategies. In addition, the use of entirely soft materials holds great promise for improving operational safety and biocompatibility (*50*) while also requiring further innovation in the integration of biocompatible sensing elements with compliant actuators.

Here, we introduce SeroTab, a sensorized ingestible soft robotic minitablet designed to enable simultaneous pH sensing and liquid sampling for prediagnostics and biomarkers collection at precise locations within the GI tract. SeroTab’s sliding locomotion, inspired by the movement of penguins, is facilitated by the optimized interaction between an external magnetic field and the embedded ferromagnetic material within the device. The liquid sampling function, enabled by a shape memory polymer (SMP), is remotely activated using an external radio frequency (rf) heater operating at a body-safe temperature. A pH-sensitive hydrogel component, engineered with a tailored structure to create a pronounced acoustic impedance contrast, enhances its detectability via US imaging. Simultaneously, the hydrogel absorbs and retains gastric fluid, preserving the sample for biomarkers analysis after retrieving. We evaluate SeroTab’s locomotion, sampling, and sensing capabilities in lab-designed setups and ex vivo swine organs. Real-time gastric pH data are successfully acquired in rabbit models in vivo. Biomarkers are extracted from the retrieved hydrogel after operation, and metabolomic analysis is conducted on the basis of the mass spectrometry analysis. The results demonstrate SeroTab’s ability to perform both rapid and precise noninvasive gastric diagnostics.

## RESULTS

### SeroTab design, functions, operating principle, and envisioned medical application

**Fig. 1.**
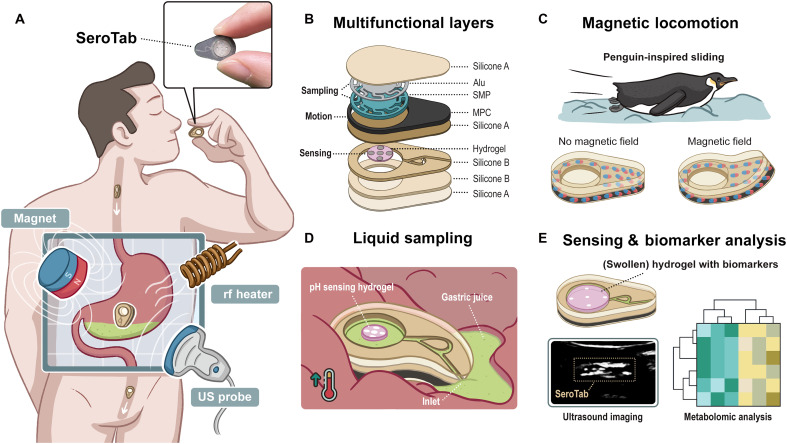
Sensorized ingestible soft robotic minitablet (SeroTab) for on-demand pH detection and biomarker collection of gastric juice. (**A**) Ingestion process and magnetic navigation of SeroTab to a targeted stomach site, followed by heat-triggered gastric juice sampling and US-assisted pH sensing. (**B**) Exploded-view schematic illustrating the device’s multimaterial composition, featuring an internal channel-cavity structure and three key functional components: (**C**) MPC layer enabling the penguin-inspired magnetically actuated locomotion. (**D**) Alu-SMP layer for heat-activated liquid pumping. (**E**) Hydrogel-based US-readable structure designed for on-site pH sensing and postanalysis of biomarkers. Illustrations created by the authors using Adobe Photoshop and Adobe Illustrator.

The SeroTab, featuring a soft, biocompatible, and wirelessly actuated design, serves as an ingestible robotic platform for noninvasive access to gastric environments (Fig. 1A and fig. S1). The device is designed for comprehensive physiological assessment at targeted gastric sites, enabling early screening and accurate diagnosis of gastric disorders. The detection process is divided into two stages: on-site pH sensing of gastric juice for rapid prediagnosis, followed by on-demand gastric fluid sampling for precise mass spectrometry analysis after retrieval (fig. S23). Targeted locomotion is achieved through external magnetic navigation guided by US imaging. Upon reaching the desired gastric site, an external rf heater induces localized heating within the device to initiate gastric fluid sampling. The pH-sensitive hydrogel embedded in the device swells upon contact with the sampled fluid, and its mechanical response is subsequently visualized via US imaging, enhanced by the inclusion of acoustic contrast agents within the hydrogel matrix (Fig. 1A). The hydrogel exhibits effective pH responsiveness across the range of 2 to 7, achieving a maximum swelling ratio of up to 62% within the SeroTab. Following the pH sensing phase, the SeroTab is retrieved from the body, and the hydrogel is extracted for mass spectrometry analysis to enable precise disease diagnosis.

Figure 1B illustrates the structure of SeroTab through an exploded view, highlighting its planar-stacked architecture that enables the integration of multifunctional components within a compact volume. At the core of the device lies a hollow cylindrical chamber (38.5-μl capacity) designed for fluid collection, which is accessed through a microfluidic channel incorporating a Tesla valve to promote unidirectional flow and minimize backflow or leakage (fig. S12). A hydrogel sensor that swells in response to pH is placed inside the chamber and embedded with metal discs to enhance acoustic visibility and enable real-time dimensional tracking via US. The chamber is sealed with thin silicone rubber membranes on both faces to prevent fluid escape. A cylindrical cavity on top hosts an SMP actuator, functioning as a sampling pump, with a conformal aluminum layer adhered on top to enable localized heating. Above the housing layer, a silicone rubber sheet embedded with magnetic particles facilitates external magnetic actuation. All layers are seamlessly integrated using a layered printing fabrication approach (fig. S2), ensuring robustness, biocompatibility, and watertight sealing suitable for gastric deployment.

The navigation of SeroTab within the body, after being swallowed, is facilitated by an external magnet. Its sliding locomotion—bioinspired by the streamlined gliding motion of penguins (Fig. 1C)—is enabled through magnetic actuation, allowing the device to navigate anatomical obstacles along tissue surfaces in a wireless, untethered manner. To enable this, the device undergoes magnetization by being fixed onto a custom fixture and exposed to a pulsed high-intensity magnetic field, resulting in a programmed magnetization profile within the magnetically responsive magnetic polymer composite (MPC) layer (fig. S2K). In the absence of an external magnetic field, SeroTab remains in a relaxed, planar state. Upon exposure to a magnetic field generated by a permanent magnet (50 to 250 mT), the device aligns with the field and bends due to the magnetic torque acting on the MPC layer (Fig. 1C). By applying a magnetic force via a moving magnet, translational motion is generated, enabling wireless and untethered sliding locomotion.

Before oral administration, SeroTab must undergo a training process to precondition the SMP and enable its fluidic sampling function. This involves placing the device on a heating plate and applying mechanical pressure to the SMP surface once it has been heated above its thermal transition temperature (40°C). The pressure is maintained as the SMP cools to room temperature (20°C), thereby fixing it in a compressed, temporary shape and sealing the internal chamber. Upon subsequent exposure to rf heating, the SMP recovers its original configuration, resulting in a rapid volume expansion (up to 38.5 μl within 1 min, depending on the applied temperature) and a corresponding drop in internal chamber pressure. The resulting pressure differential passively draws GI fluid into the chamber through the microfluidic inlet, initiating the sampling process (Fig. 1D).

Upon contact with the sampled gastric fluid, the pH-responsive hydrogel sensor undergoes a reversible volumetric swelling transition while simultaneously absorbing the surrounding liquid (Fig. 1E, top). The hydrogel is embedded with an array of biocompatible metal disks to maximize acoustic impedance mismatch with the surrounding media, thereby enhancing contrast in US imaging; their interspatial displacement, modulated by the swelling ratio, is captured in real time using an external US imaging system. This enables noninvasive, quantitative assessment of local pH variations within the GI environment (Fig. 1E, bottom left). After the sensing phase, SeroTab is retrieved from the body with gastric fluid retained in the hydrogel matrix. The collected sample is then subjected to high-resolution mass spectrometry, enabling comprehensive molecular profiling for biomarker identification and diagnostic evaluation (Fig. 1E, bottom right).

### Demonstration and characterization of magnetically actuated locomotion from 3D-printed models to ex vivo and in vivo assessments

The GI tract exhibits a highly dynamic and anatomically complex structure, characterized by distinct mucosal folds, peristaltic motion, and a mucus layer that can either facilitate lubrication and induce adhesion (fig. S7). These factors present substantial physical challenges for the locomotion and functionality of ingestible soft robotic devices. To overcome friction and mucus-related resistance during locomotion, effective control requires sufficient flexibility and actuation force, with reduced contact area serving to minimize drag. In this section, we demonstrate the advantages of SeroTab’s penguin-inspired sliding motion pattern in terms of maneuverability, transitioning from lab-designed models to ex vivo and in vivo experiments.

**Fig. 2.**
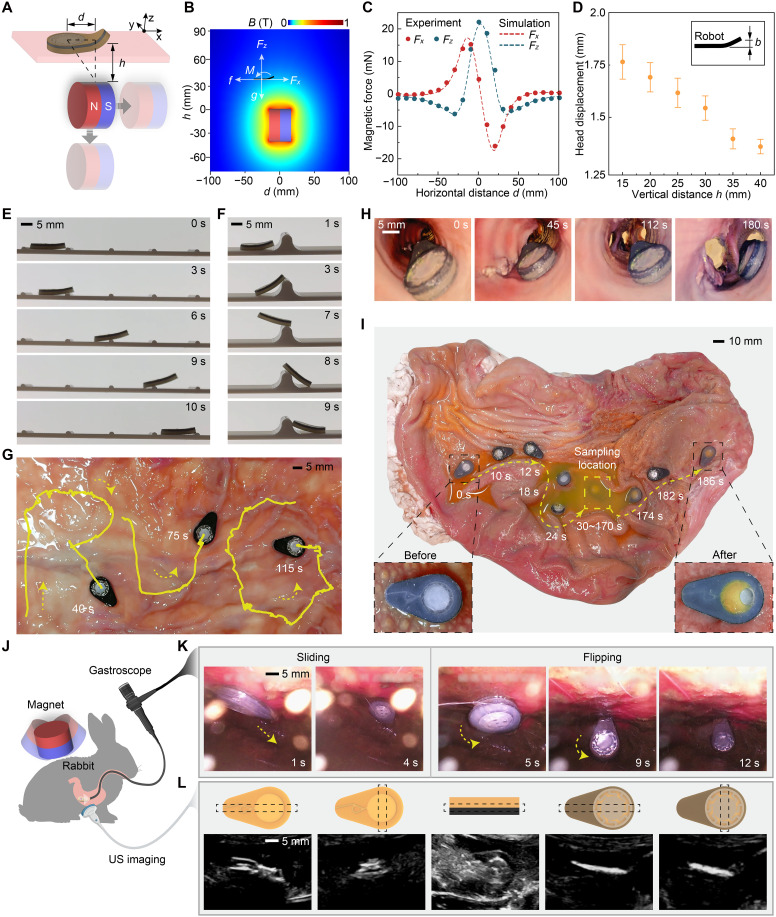
Magnetically actuated locomotion for targeted liquid sampling. (**A**) Schematic illustration of the magnetic actuation mechanism and locomotion principle. (**B**) Simulated magnetic field distribution map and magnetic force analysis. (**C**) Comparison of the measured and simulated pulling force ($F_{x}$) and lifting force ($F_{z}$) at a 15-mm vertical distance. (**D**) Assessment of SeroTab’s head-lifting displacement. (**E**) Demonstration of SeroTab’ ability to overcome small obstacles and (**F**) big obstacles (movie S2). (**G**) Maneuverability tests of sliding motion on tissue surface (movie S3) and (**H**) a tubular organ ex vivo (movie S4). (**I**) Targeted motion and liquid sampling on an opened porcine stomach (movie S5). (**J**) Schematic representation of in vivo testing setups. (**K**) Endoscopic observation (movie S8) of SeroTab’s movement to assist with (**L**) visualization using a US imaging system (movie S9) (for comparative purposes, endoscopy is used exclusively in this phase of the experiment alongside US imaging). Illustrations created by the authors using Adobe Illustrator.

The wireless actuation of SeroTab relies on the interaction between the magnetic powder embedded in its body and an external permanent magnet. To quantitatively evaluate the magnetic force and torque acting on SeroTab, we developed a force-sensing platform with two embedded force transducers to measure forces in the pulling (*x*) and lifting (*z*) directions (Fig. 2A). A cylindrical permanent magnet (N35, 30 × Φ 45 mm) is mounted underneath allowing for the adjustment in the horizontal (*d*) and vertical (*h*) directions. The relative distance between the magnet and SeroTab determines the magnetic force and torque acting on SeroTab. Centered on the midpoint of SeroTab (fixed on the platform), we move the magnet from left to right over a 200-mm distance while varying the vertical distance from 15 to 40 mm (scan area shown in fig. S5A). Force data were recorded at intervals of 10 and 5 mm per step, respectively.

We also conducted simulations using COMSOL Multiphysics (version 6.3, COMSOL AB, Sweden) to analyze the SeroTab-magnet interaction. The magnetic torque (**M**) induces bending of SeroTab’s body (eq. S2), whereas the magnetic force (**F**) consists of a pulling component ($F_{x}$), which overcomes surface friction (*f*), and a lifting component ($F_{z}$), which counteracts gravity (*g*) (Fig. 2B and eq. S1). Magnetic field distribution maps (fig. S5A) are generated to visualize the scanned area used for calculating the magnetic field (Fig. 2B and fig. S5B) and magnetic force (Fig. 2C and fig. S5C) at varying horizontal and vertical distances. The magnetic actuation generates a maximum pulling force of 22 mN and a peak lifting force of 17 mN, offering application-dependent versatility in force direction. Higher pulling force facilitates sliding locomotion by overcoming friction and adhesion, whereas greater lifting force counteracts gravity and enables flipping of SeroTab. In addition, magnetic torque induces bending of SeroTab’s body, contributing to reduce contact area. Alternative configurations using multiple magnets to enhance locomotion are presented in figs. S4 and S5.

The bending of SeroTab’s body results in the elevation of its head from the ground. Depending on the magnet’s distance, the head can be lifted by up to 1.75 mm (Fig. 2D and movie S1). To demonstrate smooth sliding motion facilitated by body bending, a 3D-printed setup with 0.5-mm-high obstacles is created (Fig. 2E and movie S2). SeroTab overcomes the surface undulations using only the linear movement of the magnet without requiring orientation adjustments, enhancing operational practicality for clinical applications. For navigating over larger obstacles, we rotate the magnet while moving it to generate higher torque, enabling SeroTab to lift or even flip (Fig. 2F and movie S2). Ex vivo experiments on tissue surfaces (Fig. 2G and movie S3) and within tubular organs (Fig. 2H and movie S4) taken from a pig demonstrate the controllability of the penguin-inspired sliding motion. A full procedure was also tested in a dissected porcine stomach containing residual gastric juice in a central pit. Starting with an empty chamber, SeroTab flips and slides into the pit, overcomes obstacles, samples the gastric juice via an rf heater, and then flips out, stopping at the target site with the chamber filled (Fig. 2I and movie S5).

In vivo validation of magnetically controlled locomotion was performed in the stomach of a live rabbit (Fig. 2J). SeroTab was administered under light anesthesia by injecting it into the esophagus with saline, enabling voluntary swallowing before deep sedation. A handheld external permanent magnet, positioned above the skin near the stomach, was used to guide and anchor SeroTab at the desired location. We also used an endoscope to visualize and confirm its correct positioning, observing both sliding and flipping motions (Fig. 2K and movie S8). Following endoscopic verification, we used a US imaging system (EPIQ7c, Philips, The Netherlands) to monitor SeroTab’s movement and status within the stomach. It should be noted that endoscopy was used only during this phase of the experiment for comparative assessment. It is not required in other stages as the capsule can be visualized and tracked using US alone. A reference table of US images corresponding to different orientations of SeroTab is created to assist users in identifying its postures (Fig. 2L). The table includes US images of the long-axis and short-axis views of Face A, the long-side edge, and the long-axis and short-axis views of Face B, arranged from left to right.

### Design and characterization of an rf heating-triggered liquid sampling actuator

**Fig. 3.**
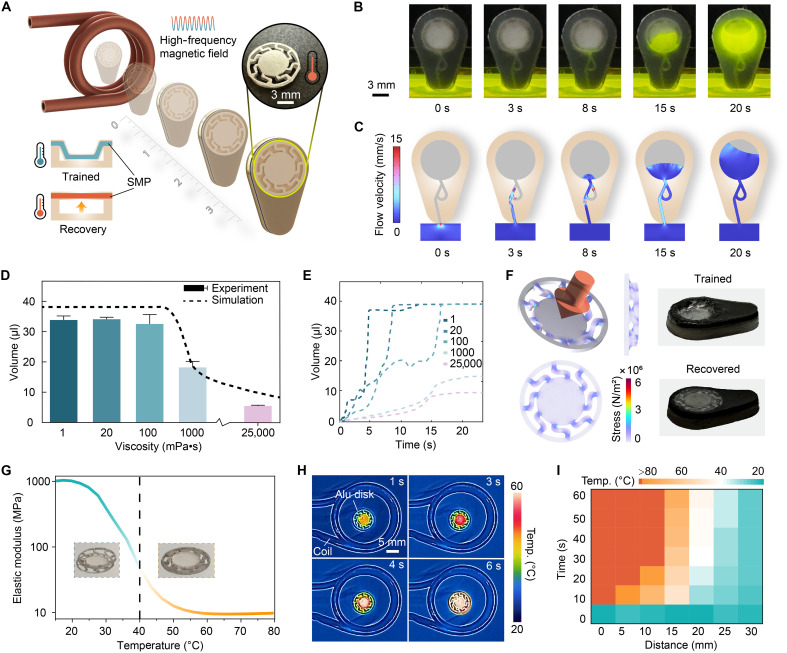
Heat-triggered liquid sampling function. (**A**) Schematic illustration of SeroTab exposed to rf heating at different distances. The built-in SMP actuator begins to recover upon reaching its transition temperature. (**B**) Experimental (movie S6) and (**C**) simulated results from sampling the fluorescent liquid. (**D**) Experimental and simulated maximum sampling volume at varying liquid viscosities. (**E**) Simulated sampling rate at different liquid viscosities. (**F**) Stress distribution of the trained SMP actuator and experimental images of SeroTab during shape training and recovery. (**G**) Temperature-dependent elastic modulus of the SMP actuator, with a transition temperature of 40°C. (**H**) Infrared thermal images of the aluminum heating element positioned 10 mm above the rf heater coil, illustrating heat transfer and distribution. (**I**) Spatiotemporal characteristics of the temperature rise in the heating element with a 0.45-kW rf heater. Illustrations created by the authors using Adobe Illustrator.

After reaching the targeted locations within the stomach through magnetic navigation, SeroTab initiates the sampling process in response to an external rf heater (SH-2/350, UltraFlex, USA) with a rated power of 3.4 kVA and an operating frequency of 125 kHz. The embedded aluminum element heats up in response to the external rf heater applied at a distance (Fig. 3A). The generated heat is transferred to the SMP positioned beneath the aluminum element. The SMP, designed with a specific planar structure to facilitate axial deformation, is positioned on the top of the chamber and functions as a pump to absorb liquid through a microchannel. Once the SMP reaches its transition temperature, it begins recovering from its trained/compressed state (Fig. 3A, bottom left). The recovery of the SMP induces a volume change in SeroTab’s chamber, resulting in a decrease in air pressure inside. When the inlet of SeroTab is placed in liquid, the pressure difference drives the liquid to flow into the chamber through a microchannel that connects it to the external environment.

To visualize the liquid sampling process, we positioned the inlet of SeroTab in a tank filled with green liquid (a green chemiluminescent solution composed of hydrogen peroxide, oxalate ester, and fluorescent dye). The rf heater, placed 10 mm behind SeroTab, continuously heats the aluminum heating receiver, triggering the recovery of the SMP. As the SMP restores its shape, the glowing liquid is drawn into the chamber (Fig. 3B and movie S6). We simulated the pressure-induced sampling system based on characterized data (figs. S10, S15, and S16) considering capillary effect (fig. S14) and the Tesla valve (fig. S12) in COMSOL Multiphysics (version 6.3, COMSOL AB, Sweden). The simulation results (Fig. 3C) closely match the experimental data, validating the accuracy of our theoretical model (eq. S6). To ensure active sampling at the desired location and minimize contamination, the capillary effect is studied and mitigated through the design and materials of the microchannel (fig. S14). In addition, an optimized Tesla valve is incorporated in the middle of the microchannel to enhance the diodicity of the fluid flow, preventing the sampled liquid from unintentionally leaking out of the chamber during operation [e.g., due to impact load from an accidental fall (fig. S13 and movie S11)]. The optimized diodicity improves as the Reynolds number of the liquid increases (fig. S12).

We further evaluated the liquid sampling capability across different viscosities. Sodium alginate solutions with varying viscosities were prepared by adjusting the weight ratio of sodium alginate to water. SeroTab successfully absorbed up to 35 μl of liquid, with a maximum viscosity capacity of 100 mPa·s (Fig. 3D). Note that the designed chamber volume of SeroTab is 38.5 μl, and the viscosity of human gastric juice ranges from 1 to 10 mPa·s. The sampling efficiency declines when the liquid viscosity exceeds this threshold. The ability to sample viscous liquids depends on the recovery performance of the SMP, which can be adjusted for specific sampling scenarios (e.g., mucus) (*51*). In addition, we simulated the time-dependent sampling process for liquids of different viscosities (Fig. 3E) based on the measured SMP recovery rate (fig. S10) at a given temperature.

The lattice structures connecting the outer ring and inner disk of the SMP enable deformation along its axial direction, transforming it from its original planar shape. The training of the SMP was performed by heating it above its transition temperature, followed by applying and maintaining a distributed force on the inner disk. We analyzed the structure mechanics of the procedure shown in Fig. 3F. Images of SeroTab in both its trained and recovered states were also captured. In our study, the transition temperature of the SMP was set to 40°C by controlling the ambient temperature during the curing process. The temperature-dependent elastic modulus was experimentally characterized for a range of temperatures using a dynamic mechanical analyzer (Viscoanalyser VA2000, Metravib) through tensile testing (Fig. 3G).

Remote heating using alternating magnetic fields offers advantages in controllability, targeting, and noninvasiveness compared to other remote heating methods such as thermochemical, photothermal, and acoustic heating (*52*). To achieve an efficient heating process, we used the Joule heating mechanism, where a high-frequency magnetic field induces eddy currents within the material. A 10-μm aluminum membrane, cut into the same shape as the SMP, was attached to it to generate and transfer heat to the SMP’s lattice structures. Heat was primarily generated at the center of the membrane and then transferred to the edges through the lattice structures (Fig. 3H). To enhance heating efficiency while maintaining structural flexibility, an additional aluminum circular disk can be placed at the central top of the shaped aluminum membrane. We investigated the heating performance as influenced by key variables, including the rf heater’s output power, material dimensions, operating distance, and heating duration (fig. S8). Our results indicate that sufficient heating (40°C) can be achieved within 30 s at distances of up to 2 cm (Fig. 3I), which is considered adequate for applications in the rabbit stomach in this study. Larger heating distance can be readily achieved by increasing output power or using a thicker aluminum disk, thereby extending potential applications to deeper organs or within the human body as discussed in the Supplementary Materials.

### Characterization and calibration of an acoustic pH sensor

**Fig. 4.**
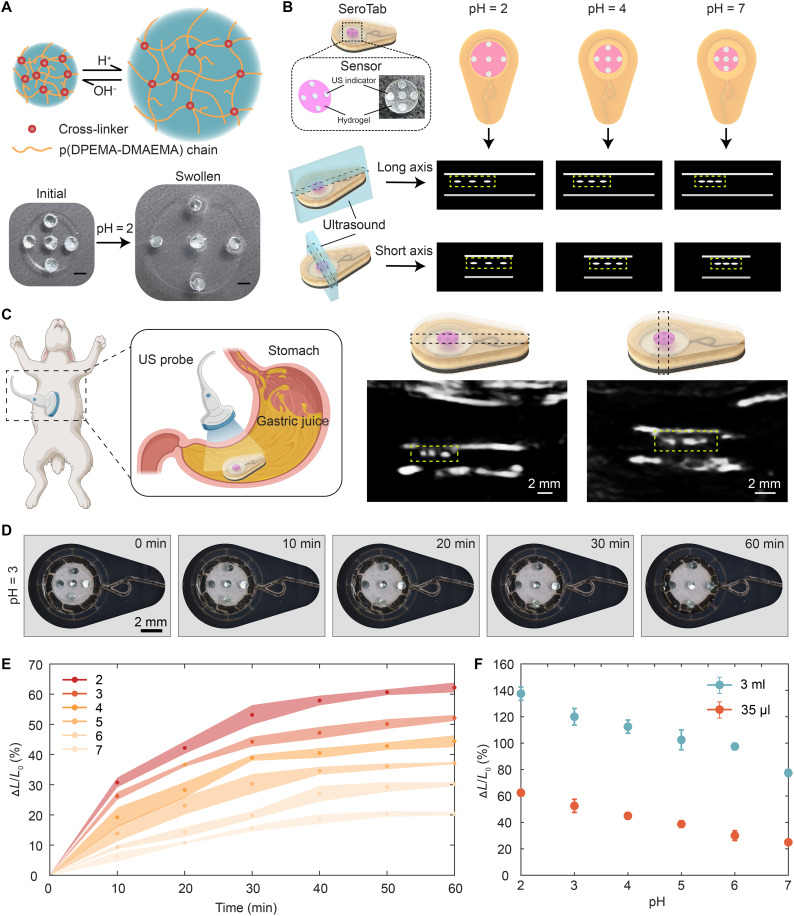
Hydrogel-based pH sensor for ultrasonic detection. (**A**) Schematic illustration (top) and experimental demonstration (bottom) of the pH-responsive hydrogel swelling mechanism. Scale bars, 1 mm. (**B**) Integration of the pH sensor within the SeroTab chamber. The distributed zinc disk inside the hydrogel enhances ultrasonic contrast, enabling detection of hydrogel size changes in response to different pH solutions. (**C**) In vivo verification of ultrasonic detection of SeroTab and the pH sensor in a live rabbit stomach. US images of the long axis (left) and short axis (right) are captured. (**D**) Time-dependent response of a SeroTab when immersed in simulated gastric juice at pH 3 (movie S7). (**E**) Calibration of time-dependent responses of the pH sensor in a 35-μl chamber with solutions of different pH values. $\Delta L/\Delta L_{0}\left(\%\right)$ denotes the swelling ratio. (**F**) Saturated swelling ratio of the pH sensor after immersion in 35 μl and 3 ml of gastric juice at different pH values for 12 hours. Illustrations created by the authors using Adobe Illustrator.

We integrated a pH-responsive sensor into the chamber of SeroTab to enable real-time chemical monitoring upon command. The sensor consists of a pH-sensitive hydrogel that undergoes swelling in response to the surrounding fluid (Fig. 4A, top), producing a mechanical expansion that can be quantified by US imaging through the integrated high US contrast metal disks (Fig. 4A, bottom). To achieve this functionality, we adapted a hydrogel formulation previously developed for implantable US-readable sensing applications (*41*) and incorporated it into our magnetically actuated robotic platform. The hydrogel was synthesized using p(DMAEMA-DPAEMA) (poly[2-(dimethylamino)ethyl methacrylate-*co*-2-(diisopropylamino)ethyl methacrylate]), with polyethylene glycol diacrylate (PEGDA; number-average molecular weight *M_n_* = 700) as a cross-linker. DMAEMA and DPAEMA contain tertiary amine moieties that undergo protonation with decreasing pH, leading to subsequent changes in osmotic pressure. To balance the need for both rapid response time and compact dimensions, the hydrogel was cut into circular disks with a diameter of 4 mm and thickness of 200 μm. To enable US visualization of hydrogel expansion, five circular Zn disks (1 mm in diameter and 50 μm in thickness) were embedded within the hydrogel to create a high acoustic impedance mismatch. The design of the hydrogel and the distribution of the Zn disks are illustrated in (fig. S3F). Figure 4A (bottom) presents experimental observations of the sensor dimension before and after swelling in a simulated gastric juice at pH = 2.

US B-mode imaging is used to quantitatively evaluate the pH-dependent geometry of the hydrogel sensor while simultaneously tracking and observing the entire SeroTab, where the sensor is embedded. The acoustic impedance mismatch between the Zn disks and surrounding materials—including the hydrogel matrix, gastric juice, tissue, and silicone rubber—enhances their visibility in B-mode images. During a US scan, acoustic waves generated by the transducer penetrate tissues and reflect off the SeroTab and Zn disks, forming cross-sectional images that reveal their spatial relationships. In the specific hydrogel sensor design presented here, a Zn disk was positioned at the center of the hydrogel piece, with additional Zn disks symmetrically placed on either side. The cross-sectional positioning of these disks enables ultrasonic measurement of the sensor’s diameter. Their symmetric and circular distribution allows for orientation-independent ultrasonic visualization. Figure 4B illustrates the concept of US imaging along the long and short axes of SeroTab from the view of its Face A, demonstrating how the distances between Zn disks change with varying pH levels. In the US images, the three bright dots represent the cross-sectional views of the Zn disks, whereas the bright lines correspond to the upper and lower surfaces of SeroTab.

We evaluated the feasibility of ultrasonic detection for pH sensing in a live rabbit model. The US scan began after gastric juice was sampled into SeroTab’s chamber, where the sensor is located. The position and orientation of SeroTab within the stomach can be identified from US images acquired from different scanning directions as shown in Fig. 2L. A magnet was used to adjust and fix the orientation of SeroTab, ensuring that Face A was aligned with the US probe so that the Zn markers embedded in the hydrogel sensor became visible. Successful measurements are indicated by the appearance of three bright spots at equal distance (fig. S17A), whereas fewer than three visible spots indicate an invalid measurement (fig. S17B), requiring further adjustment via magnetic control and US probe reorientation. By adjusting the US system’s output parameters (e.g., dynamic range, gain, frequency, and focus), we enhanced the acoustic visibility of the Zn disks while minimizing noise. SeroTab and Zn disks were successfully visualized across the sensor’s diameter, and US images along both the long and short axes are captured (Fig. 4C).

To calibrate the relationship between the mechanical response of the hydrogel sensor and the pH level of the surrounding solution, we used simulated gastric fluid (SGF) with pH values ranging from 2 to 7. Figure 4D and movie S7 represent experimental measurements for the time-resolved swelling behavior of the sensor in the cavity of SeroTab filled with 35 μl of SGF solution at pH = 3. Upon direct exposure to SGF at low pH (e.g., pH = 2), the sensor swells by 30% in less than 10 min and reaches more than 60% in 60 min. As shown in Fig. 4E, the swelling ratio ($\Delta L/\Delta L_{0}$) exhibits a continuous increase over 60 min, whereas it decreases with increasing pH. In a larger volume of SGF (3 ml), the sensor reaches its saturated swelling state (up to 135% at pH = 2) after 12 hours (Fig. 4F), indicating its capacity for prolonged exposure and potential to sample and sense larger volumes of gastric fluid.

### In vivo study

**Fig. 5.**
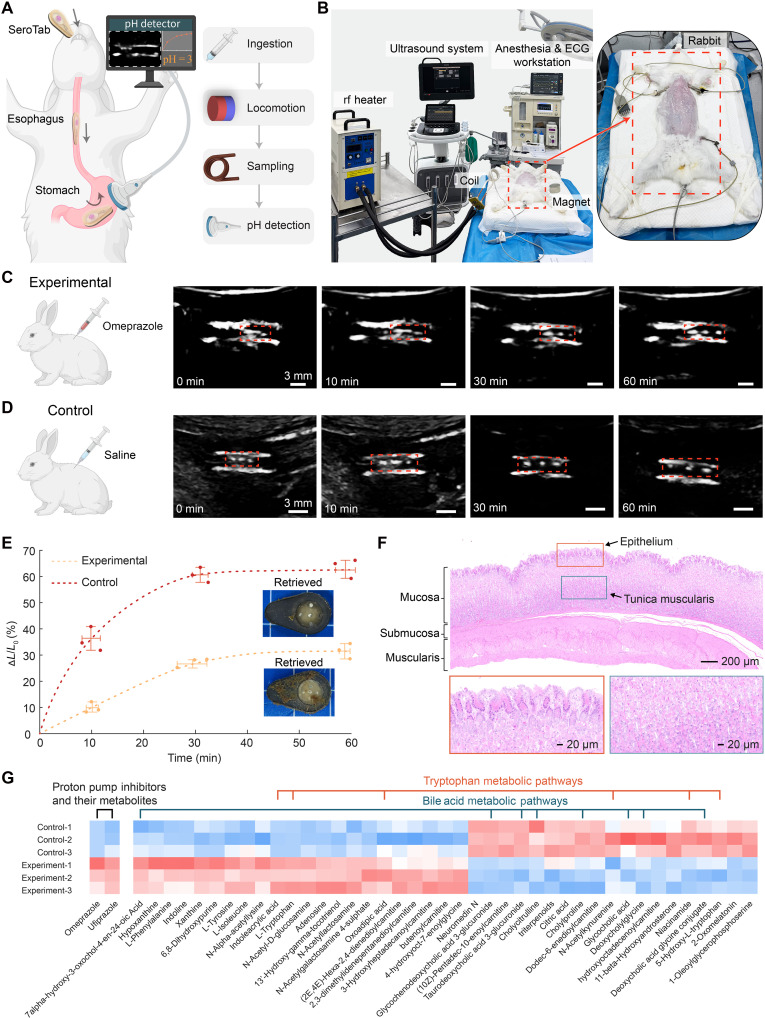
In vivo study: In situ pH detection and postsampling biomarker analysis of rabbit gastric juice. (**A**) Schematic illustration and (**B**) experimental setups of the oral administration, targeted sampling, and sensing procedure for a SeroTab in the rabbit stomach. ECG, electrocardiogram. (**C**) US images (right) of the SeroTab and sensor in the rabbit stomach from the experimental group and (**D**) control group, highlighting the expansion of the pH sensor (movie S10). (**E**) Summary data showing the swelling ratio of the pH sensor in both experimental and control groups (*n* = 3 independent samples per group). (**F**) H&E staining of gastric cross sections demonstrates the biocompatibility of SeroTab. (**G**) Results of mass spectrometry and metabolomic analysis. Illustrations created by the authors using Adobe Illustrator.

We performed in vivo live rabbit experiments in which we comprehensively test SeroTab’s feasibility and operability for oral delivery, magnetic control, sampling, pH sensing, and biomarker analysis. The schematic and experimental setups are presented in Fig. 5 (A and B), respectively. SeroTab was positioned at the oropharynx and swallowed into the rabbit’s stomach with the aid of a 5-ml physiological saline flush. US scans were used to confirm gastric entry, following which magnetic navigation was used to guide and position SeroTab in the middle part of the stomach (movie S9). Remote heating was then applied via the rf heater to initiate sampling of the surrounding gastric fluid. The heating distance was maintained at ~2 cm, with an activation duration of 30 s to achieve a transition temperature of 40°C (fig. S8). The US probe was fixed in place throughout the swelling procedure to visualize a consistent cross section of the sensor, where three bright dots (corresponding to embedded Zn markers) were observed. US images were recorded, and the distances between the markers were measured at defined time points (0, 10, 30, and 60 min; movie S10).

In this study, we explored a potential application of SeroTab as part of clinical diagnostics for gastric physiology by demonstrating its use in detecting gastric pH. We used omeprazole, which is a proton pump inhibitor (PPI) primarily used to treat conditions caused by excessive gastric acid secretion, such as gastric ulcer and GERD. Its administration leads to an elevation of gastric pH by inhibiting the H^+^/K^+^–adenosine triphosphatase (ATPase) in parietal cells, thereby reducing acid secretion. We injected omeprazole subcutaneously (20 mg/kg) 3 hours before anesthesia to regulate gastric acid secretion in live rabbit models (*n* = 3 animals, experimental). To confirm model establishment and enable comparison, gastric juice samples were collected via gastric tube, and their pH values were measured externally before the operation. SeroTab was delivered postanesthesia to sample gastric juice from the stomach. US images of the hydrogel sensor inside SeroTab were obtained at 0, 10, 30, and 60 min (Fig. 5C), and the swelling ratio of the sensors were measured (Fig. 5E). The corresponding pH values were inferred from the measured time-resolved swelling ratios by referencing the calibration curve shown in Fig. 4E. For comparison, the same procedure was repeated using saline as a substitute for omeprazole (Fig. 5D). Following the pH sensing procedure, SeroTab was retrieved from the rabbit stomach. To assess its biocompatibility and safety, tissue samples from the gastric operation site were collected, processed for hematoxylin and eosin (H&E) staining, and subjected to histological analysis under microscopy (Fig. 5F and fig. S21). The results showed no evidence of epithelial disruption, inflammatory infiltration, or necrotic damage in the gastric mucosa, indicating good tissue compatibility and thermal safety of the SeroTab during operation.

In the current test protocol in the live rabbits, the complete SeroTab operation, including SeroTab ingestion, locomotion, sampling, and sensing, requires ~70 min. The hydrogel swelling (until saturation) accounts for most of this duration (about 60 min), although distinct volumetric changes sufficient for reliable pH determination can be observed within the first 10 min. Accordingly, the estimated operational duration of a clinically optimized version is expected to be under 20 min, which is comparable to standard GI endoscopy examinations (*53*). Further reduction of operation duration through optimization of hydrogel response (as described in the Supplementary Materials) and operation automation can improve patient outcomes.

To evaluate the molecular diagnostic potential of SeroTab, we conducted high-resolution mass spectrometry–based nontargeted metabolomic analysis on the hydrogel matrix retrieved from the device following in vivo gastric fluid sampling. The analysis identified 43 metabolites with significant changes (*P* value < 0.05) and a fold change (FC) exceeding ±1.5 between experimental groups, as determined by MS^2^ spectra. Among these, the omeprazole component and 42 associated metabolites were detected, including 12 amino acids and 8 bile acid–related compounds. These findings underscore the hydrogel’s ability to efficiently sequester and preserve gastric biomolecules, enabling robust downstream profiling. Further pathway analysis revealed that omeprazole administration induced notable alterations in bile acid metabolism and tryptophan metabolism pathways—two key metabolic axes intimately associated with gastric acid regulation and gut microbiota dynamics (Fig. 5G). These results are consistent with omeprazole’s pharmacodynamic mechanism, wherein inhibition of the H^+^/K^+^-ATPase in parietal cells elevates gastric pH, consequently disrupting the intestinal microbial environment and suggesting a significant impact on host-microbiome interactions. Moreover, the detected metabolite clusters can serve as potential biomarkers for detecting drug-associated adverse effects, monitoring therapeutic outcomes, and guiding personalized treatment strategies in patients receiving PPI therapy.

These findings not only demonstrate the biochemical fidelity of SeroTab’s hydrogel-based sampling system, but also highlight its value as a noninvasive platform for in situ diagnostics within the GI tract. Given that changes in gastric fluid composition have been implicated in various GI diseases, this approach holds promise for enabling both rapid prediagnosis and precise molecular analysis for various gastric diseases. In particular, disease states such as gastric cancer are known to induce distinct shifts in gastric secretions and metabolite concentrations, highlighting the potential of this method for early-stage, noninvasive disease screening and personalized therapeutic monitoring.

## DISCUSSION

This study demonstrates the utility of SeroTab in enabling both rapid and precise examination of gastric physiology by integrating a soft robotic tablet with a built-in pH-sensitive hydrogel, supported by conventional US imaging and mass spectrometry analysis. Both ex vivo and in vivo tests demonstrate consistency in pH detection and sampling compared to traditional gastric tube aspiration, whereas our device offers advantages in flexibility, miniaturization, and wireless operation, highlighting its potential to access challenging anatomical regions, such as the duodenum and small intestine, with reduced patient discomfort. This study demonstrates a fully integrated soft robotic system capable of magnetically controlled movement, on-demand gastric juice sampling, and real-time pH sensing. All functions are demonstrated in animal models in vivo, indicating SeroTab’s ability to detect pharmacologically induced pH changes and metabolic shifts following omeprazole administration. The penguin-inspired robotic design, actuated by external magnetic fields, allows the device to adaptively navigate anatomical obstacles and reach targeted gastric regions. The proposed multilayered printing technique enables the integration of multiple functional materials, including a thermally responsive SMP, into a compliant soft body, achieving large-volume liquid sampling through remote rf heating. The use of a flexible, biocompatible hydrogel allows both US-based pH sensing and preservation of biomarkers for mass spectrometry analysis, an integration not reported in previous soft robotic systems. It addresses the incompatibility between rigid components and soft robotic systems, enhancing both biocompatibility and flexibility.

The integration of in situ pH sensing and on-demand gastric fluid sampling provides a complementary diagnostic approach. Because fluctuations in gastric pH are closely associated with multiple GI disorders such as gastric ulcer, gastritis, and infection, real-time in situ sensing can help determine whether subsequent molecular analysis is necessary. Postanalysis of the retrieved hydrogel offers detailed biochemical and molecular information for definitive diagnosis (fig. S23). This combined strategy establishes a balanced diagnostic workflow that supports both rapid screening and precise follow-up analysis, potentially improving clinical efficiency and reducing unnecessary procedures.

In contrast to conventional cylindrical ingestible robotic devices such as capsule endoscopes, SeroTab features a planar-stacked architecture that enables a space-efficient design. This configuration accommodates multiple functional layers within a compact volume while potentially reducing swallowing difficulty and lowering the risk of GI obstruction (*54*, *55*). The layered printing fabrication method enables the precise construction of complex structures, such as the microfluidic channels with a Tesla valve, and the integration of multiple materials to support multifunctional capabilities. Also, it offers foundational fabrication techniques for more customized designs that produce specific motion patterns, such as swimming or crawling, to adapt to various regions and environments within the human body, including the vascular and intestinal systems. However, further miniaturization of the device is necessary to enable navigation through highly confined anatomical regions, such as neural networks and urinary tracts. In such cases, US-based detection of the miniaturized hydrogel structures may become challenging due to resolution limitations. Alternative high-resolution imaging modalities, such as x-ray (fig. S19B) or computed tomography, should be considered for effective visualization.

Sampling high-viscosity biological fluids expands SeroTab’s applications. For example, mucus [20 to 1000 centipoise (cP)] from the GI tract contains critical biological data, including bacteria, immune cells, and metabolites, enabling diagnostic insights into GI diseases and microbial imbalances. This requires tuning the mechanical properties of the SMP-based pumping actuator to enhance its recovery ability. Notably, the pumping mechanism can be adapted to design a drug delivery system by reversibly training the SMP. In addition, developing hydrogels capable of sensing a broader range of pH values (*41*), or other physiological parameters such as temperature and pressure, will further expand the utility of the device across various organs and environmental conditions. Although the hydrogel used in this study is designed for single-use, short-term sensing within the gastric environment, long-term stability and potential signal drift should be systematically evaluated. In addition, further exploration of its reversible swelling and deswelling (*56*) behavior could enable future applications in continuous monitoring.

In the event of unexpected exposure or rupture in the acidic gastric environment, the magnetic composite used in the device is inherently stable, and its biocompatibility can be further improved by incorporating biocompatible and acid-resistant polymer coatings on the magnetic particles (*57*). Future designs may incorporate alternative materials such as tungsten (W) or molybdenum (Mo) as US markers in place of Zn as they are chemically stable under gastric conditions while maintaining high US contrast (*58*) to mitigate potential risks in the event of unexpected exposure or rupture in the acidic gastric environment. In addition, ferromagnetic SmFeN particles have demonstrated superior cytocompatibility compared with praseodymium-iron-boron (PrFeB) particles (fig. S22), making them a promising candidate for future developments. Collectively, these considerations highlight opportunities to integrate more biocompatible and chemically robust materials to further improve device safety and long-term clinical applicability.

In this study, magnetic control is used to guide and anchor SeroTab in the middle portion of the stomach, minimizing inter-animal variation, and allowing for consistent and reproducible gastric juice sampling and sensing. Passive locomotion, primarily driven by intestinal peristalsis, has been widely adopted in capsule devices for biomarker-triggered sampling, general diagnostics, and screening (*19*, *35*, *39*, *59*). However, magnetic actuation provides superior site-specific control, allowing localized, on-demand sampling at the target region and enabling long-term monitoring at the lesion site once detected. Other wireless actuation methods, such as optical, acoustic, or chemically actuated systems, represent potential alternatives but require careful evaluation of actuation power, biocompatibility, and response time.

The rf-triggered actuator in SeroTab enables active sampling that is decoupled from magnetic control and independent of the local biochemical environment, in contrast to osmotic sampling capsules that rely on passive diffusion or specific biochemical triggers to initiate collection (*31*, *37*) while also delivering high suction pressure, rapid response, and the capability to sample highly viscous fluids (up to 1000 mPa·s) (*30*). Future designs aiming for diagnostic specificity could incorporate smart, disease-responsive actuators that autonomously trigger upon specific biomarkers (e.g., abnormal pH, enzymatic activity, or pathogenic presence) (*35*, *59*), thereby eliminating the need for external heating devices and improving system portability and clinical accessibility. However, these should account for disease-induced physiological variations (such as pH fluctuations caused by GERD) and should ensure that actuation is not triggered prematurely.

Despite addressing the challenge of integrating sensing and actuation within a single compact device and verifying its performance in the gastric environment, further studies on postgastric behavior are needed to facilitate future clinical translation. In this work, the device was retrieved by anatomical dissection to focus on functional validation in the gastric environment. Consequently, additional evaluation of structural integrity and sealing reliability during intestinal transit and natural excretion is required to minimize the risk of contamination. Future studies should focus on improving biosafety and controllability, including optimizing sealing strategies and strengthening magnetic guidance to enable reliable intestinal transit and accelerate safe excretion in the complex GI environment.

From a translational standpoint, the current prototype requires coordinated operation of magnetic actuation, rf heating, and US imaging modules for experimental validation, which may complicate clinical workflow in its present form. In future developments, these modules could be integrated into a single platform, potentially in combination with a robotic positioning system for automated operation. With computer-aided control, standardized preprogrammed operation modes, and AI-assisted image recognition, clinicians would be able to perform capsule localization, activation, and decision-making through an intuitive, simplified workflow (fig. S23). In addition, the integration of emerging wearable US technologies, such as flexible US patches (*48*), offers wireless, continuous, and high-resolution imaging for real-time localization and monitoring, with superior portability and user-friendliness compared to conventional US systems. Collectively, these advances would accelerate the clinical translation of soft robotic diagnostic platforms toward integrated, noninvasive, and patient-tailored health care solutions capable of supporting early diagnostics and real-time decision-making in primary care settings.

## MATERIALS AND METHODS

### Materials and fabrication of the SeroTab enclosure

The fabrication of the SeroTab enclosure followed a layered printing strategy that enables strong interfacial bonding between multiple functional materials (fig. S2). Laser-cut, self-adhesive polyethylene terephthalate (PET) films (100 μm thick) were patterned into four mold types based on the design specifications for each layer (fig. S3), each incorporating a rectangular contour to facilitate precise alignment during assembly (fig. S2A). Mold-1 (0.5 mm thick) was placed on a flat polymethyl methacrylate (PMMA) base, followed by casting and blade printing of silicone B (Dragon Skin 30, Smooth-On Inc., USA) (fig. S2B), which was cured at 50°C for 2 hours. Mold-2 and mold-3 (each 0.5 mm thick) were then sequentially applied, with repeated casting and curing to form internal structures. Removal of the central portions in these layers created a sampling chamber and a microfluidic channel integrated with a Tesla valve to enhance directional flow and prevent backflow (fig. S2, C and D). A prefabricated hydrogel-based pH sensor was positioned within the chamber (fig. S2E), followed by gelatin filling for temporary structural support (fig. S2F). After cooling and curing at room temperature (10 min), mold-1 (0.2 mm thick) was placed on top and sealed using silicone A (Ecoflex 00-10, Smooth-On Inc., USA) casted and cured to seal the chamber and channel (fig. S2G). Mold-4 (0.6 mm thick) was then aligned and adhered on top, followed by casting and curing of an MPC, which consists of a silicone matrix (Ecoflex 00-10, Smooth-On Inc., USA) embedded with ferromagnetic PrFeB powder (mean particle size: 5 μm; MQFP-16-7-11277, Magnequench GmbH, Germany) (fig. S2H). The mass ratio of the magnetic microparticles to the silicone rubber was 2:1 in this study. The central section of mold-4 was removed to form a cavity housing an SMP (NOA63, Norland Products Inc., USA) and a matched aluminum heating receiver (fig. S2I). A final sealing layer (mold-1, 0.2 mm thick) was added and cured to complete the structure (fig. S2J).

After curing process, SeroTab was extracted from the mold. The assembled SeroTab was then subjected to magnetization by applying a 2-T impulse magnetic field using an impulse magnetizer (ASC Model IM-10-30, ASC Scientific, USA), thereby establishing its internal magnetic profile necessary for navigation (fig. S2K). To prepare the SeroTab for use, the gelatin filling must first be discharged through the microfluidic channel by applying heat and pressure. The device was heated to 60°C, after which a cylindrical punch (6 mm in diameter) was used to compress the SMP. The training process was completed by maintaining the compression until the SMP cools down to 30°C, allowing it to fix into the deformed configuration.

### Design, fabrication, and characterization of the SMP actuator

The SMP functions as a pumping actuator, consisting of an outer ring and a central disk connected by multiple S-shaped pillars. The design and dimensions of the SMP actuator are illustrated in fig. S3E. When the inner disk was pressed while the outer ring was fixed, above the polymer’s transition temperature, a spatial displacement occurred between the two components. To accommodate the limited ductility of the SMP, the S-shaped pillars were used to provide mechanical compliance. These structures allow for relative displacement through controlled bending and twisting while maintaining overall structural integrity.

The fabrication of the SMP actuator was a two-step replica molding process. First, a positive master mold was 3D printed to define the desired geometry. This master was then used to fabricate a negative mold by casting polydimethylsiloxane (PDMS; 10:1 monomer–to–cross-linker ratio by weight), followed by curing at 50°C for 6 hours to form an inverse template. Subsequently, the SMP (NOA63, Norland Products Inc., USA) was cast into the PDMS negative mold, degassed for 5 min at 100 mbar to eliminate trapped air and ensure complete mold filling. The SMP was then preheated at 40°C, or 5 min, followed by ultraviolet A (UV-A) light exposure for 30 min at 40°C. After curing and cooling, the fully formed SMP actuator was carefully demolded.

To establish the programmed actuation profile, each SMP actuator first underwent a standard thermomechanical training procedure consisting of heating above the transition temperature, manual deformation into the target configuration, cooling to fix the temporary shape, and reheating to recover the original geometry. To evaluate the reproducibility of this training process, we performed repeated programming-fixation-recovery trials on multiple samples. Three SMP actuators were subjected to 20 consecutive training cycles, and the deformation after fixation (Δ*h*) and after recovery ($\varepsilon_{m}$) was recorded for each cycle to calculate the shape fixity $\left(\frac{\Delta h}{\varepsilon_{d}}\times 100\%\right)$ and shape recovery ratios $\left[\left(1-\frac{\varepsilon_{m}}{\Delta h}\right)\times 100\%\right]$ by comparing to the designed deformation ($\varepsilon_{d}$). As shown in fig. S11, both ratios remained consistently near 100% across all cycles for all three samples, with negligible variation either within individual samples or between different samples. The results demonstrate that the SMP actuator exhibits highly stable and repeatable thermomechanical behavior, indicating that the programmed shape memory response can be reliably maintained over extended operation.

### Synthesis and fabrication of the hydrogel-based sensor

The pH-responsive hydrogel was synthesized via a copolymerization strategy using DMAEMA and DPAEMA, both purchased from Sigma-Aldrich. The monomers were mixed in a 7:3 weight ratio (DMAEMA:DPAEMA). In the first polymerization stage, 2 wt % of the photoinitiator 2-hydroxy-2-methylpropiophenone (Darocur 1173, Sigma-Aldrich) was added to the mixture, which was then exposed to UV light (365 nm, 500 mW/cm^2^) for 1 min to initiate the formation of oligomeric chains. Subsequently, PEGDA ($M_{n}\approx 700$; Sigma-Aldrich) was added at a concentration of 1.9 wt % relative to the total monomer content to enable network formation. To facilitate the final cross-linking step, 0.1 wt % of a secondary photoinitiator, 2,2-dimethoxy-2-phenylacetophenone (DMPA; Sigma-Aldrich), was incorporated. The resulting prepolymer solution was transferred into a glass syringe for subsequent sensor molding and polymerization.

The sensor fabrication procedure followed a sandwich-like assembly method, in which two hydrogel layers were formed sequentially with a Zn disk embedded at the interface. Two hydrophobic, soft, and transparent plastic film were assembled with a 75-μm gap between them, created using a double-sided adhesive tape to form a microchannel. Each film was affixed to a glass slide to ensure a flat and uniform surface. The prepolymer solution was carefully dispensed at the entrance of the channel, and capillary action enabled the solution to spontaneously fill the gap. The assembly was then exposed to UV light for a second curing process lasting 7 min, resulting in the formation of a chemically cross-linked hydrogel matrix responsive to environmental pH changes. After curing, one membrane was peeled off, and Zn disks were positioned according to the designed layout. A new film was then placed on top, maintaining a 125-μm interfilm spacing. The capillary-driven filling and subsequent UV polymerization steps were repeated to fabricate the second hydrogel layer, encapsulating the Zn disk at the interface between the two layers. Last, individual hydrogel sensors were extracted using a 4-mm precision biopsy punch. Before use, the sensors were conditioned by immersion in phosphate-buffered saline (PBS; pH 7.4; Sigma-Aldrich) for 12 hours, followed by drying under ambient conditions.

Note that the swelling process of the hydrogel sensor until full saturation can take more than 1 hour. However, detectable change in swelling ratio occurs within the first 10 min, allowing reliable pH determination during this period (Fig. 4E). The response time can be further accelerated by reducing the hydrogel’s thickness or by tuning the copolymer composition between DMAEMA and DPAEMA. A higher proportion of DMAEMA increases the swelling ratio and decreases the response time, whereas a higher proportion of DPAEMA enhances the Young’s modulus and elongation at break. In addition, adjusting the cross-linking density (PEGDA content) can modulate the hydrogel’s response time, swelling ratio, and mechanical properties (*56*, *60*). Other strategies, such as incorporating fixed fiducial markers on the nondeformable part of the device in future designs, could provide a stable reference baseline for hydrogel deformation measurements. This configuration would reduce the number of Zn markers required within the hydrogel layer (≤3), thereby simplifying the sensor structure and fabrication process. Furthermore, minimizing the hydrogel volume could accelerate the swelling response and facilitate overall device miniaturization, ultimately improving both sensing performance and integration potential.

### Ex vivo experiments

Porcine GI anatomy and tissue properties closely resemble those of humans, making it a suitable ex vivo model for preliminary evaluation. Fresh porcine organs were obtained from a local slaughterhouse and used within 12 hours postmortem. Mucosal surfaces and residual fluids (e.g., mucus and gastric juice) were preserved to mimic in vivo conditions. For locomotion experiments, the stomach, small intestine, and trachea were used. All organs were used as received, without any additional cleaning procedures. Gastric juice was collected for sampling experiments (Fig. 2I), surface tension measurements (fig. S15), and contact angle measurements (fig. S16). Only a portion of the organs was cleaned to remove mucus for friction measurements, which were then compared with mucus-covered surfaces (fig. S7).

### In vivo experiments

Studies involving animals were conducted at Shanghai Xinova Medical Research Co. Ltd. on six healthy New Zealand White rabbits (~3.0 kg, sex not limited), obtained from a certified supplier [animal license no. SYXK (Hu) 2021-0019; approval no. XNM-YX-20241219-01]. All animal procedures were reviewed and approved by the Institutional Animal Care and Use Committee of Xinova and performed in accordance with international ethics guidelines and the National Institutes of Health Guide for the Care and Use of Laboratory Animals.

Rabbits were fasted for 48 hours before the procedure, with free access to water. Anesthesia was induced using Su-Tai (2 to 5 mg/kg, intravenously or intramuscularly) and maintained via 1 to 2% isoflurane inhalation through a veterinary anesthesia machine (WATO EX-20 Vet, Mindray, China). Animals were placed in the supine position, intubated, and continuously monitored using a multiparameter monitor (MX550, Philips, The Netherlands). Intraoperative fluid administration was provided as needed, and physiological parameters were recorded every 15 min.

Gastroscopy was performed 3 hours after omeprazole or saline injection to assess gastric conditions and collect 0.5 ml of native gastric fluid for baseline pH measurement. SeroTab was then orally administered using a small volume of physiological saline to facilitate swallowing and navigated to the targeted gastric site by an external permanent magnet. Upon reaching the site, an external rf heater was applied to activate the device’s sampling mechanism. US imaging (EPIQ7c, Philips, The Netherlands) was subsequently used to visualize the device in situ and monitor pH-responsive deformation through the displacement of embedded metal markers. In a preliminary animal study, endoscopic imaging (Fig. 2K) and x-ray imaging (fig. S19) were performed to confirm the positioning and immersion of the device within the stomach. Endoscopic imaging and x-ray imaging were used only during early trials for positional confirmation; later experiments relied solely on US guidance.

At the end of the in vivo experiment, all rabbits were humanely euthanized under deep anesthesia via intravenous injection of 10% potassium chloride (1 ml/kg), following the institutional SOP for euthanasia at Shanghai Xinova Medical Research Co. Ltd. Upon confirmation of death, a full abdominal dissection was performed. The stomach was carefully excised and opened to retrieve the SeroTab, which was gently extracted using tweezers, avoiding contact with the central sensing region. The device was rinsed, sealed in a sterile cryotube, and stored at −80°C for further analysis. Gastric tissue in contact with the device was inspected macroscopically for signs of damage (e.g., bleeding, edema, and necrosis), photographed, and fixed in 10% neutral-buffered formalin (at a volume ratio of ≥10:1) for histological analysis. Tissue sections were later processed for H&E staining and microscopic evaluation (Fig. 5F and fig. S2I). All sample collection and preservation procedures followed standardized protocols to ensure consistency and traceability.

### Metabolomic analysis

For the metabolomic profiling of gastric fluid absorbed by the hydrogel, each sample retrieved from SeroTab was homogenized in 1 ml of ice-cold methanol/water (4:1, v/v) using a bullet blender. After centrifugation at 15,000*g* for 10 min at 4°C, the supernatant was collected and subjected to vacuum drying at 4°C. The resulting residues were reconstituted in 50% methanol (methanol/water, 50:50, v/v) and analyzed using an untargeted metabolomics approach. Data acquisition was performed on a Thermo Fisher Scientific Vanquish UHPLC system coupled with an Orbitrap Exploris 120 high-resolution mass spectrometer (Thermo Fisher Scientific), following previously established protocols (*61*–*63*). Metabolite separation was carried out on an ACQUITY UPLC HSS T3 column (2.1 mm by 100 mm, 1.8 μm; Waters Corporation, Milford, MA, USA), with a mobile phase consisting of 0.1% formic acid in water (A) and 0.1% formic acid in acetonitrile (B). To capture a broader range of metabolomic features, data were acquired in both positive and negative ionization modes. Raw mass spectrometry data were processed using the Progenesis QI software (Nonlinear Dynamics, Newcastle, UK) for peak alignment, detection, deconvolution, normalization, and metabolite identification. Differential metabolite analysis was conducted by calculating the FCs between groups, with statistical significance determined on the basis of a threshold of FC > 1.5 and *P* value < 0.05. Statistical significance was determined using Student’s *t* test. The METLIN Database (http://metlin.scripps.edu/) and Human Metabolome Database (http://hmdb.ca/) were used for identification and alignment of metabolites. The KEGG (Kyoto Encyclopedia of Genes and Genomes) database was used for metabolic pathway enrichment analysis further to investigate the biological relevance of the altered metabolites.

### In vitro tests of cytocompatibility

To assess the biocompatibility of SeroTab, in vitro cytotoxicity tests were conducted using a human colorectal adenocarcinoma cell line (HT29) with epithelial morphology. HT29 cells were cultured in high-glucose Dulbecco’s modified Eagle’s medium (DMEM; Gibco) that contains 10% fetal bovine serum (FBS; Gibco) and penicillin-streptomycin (1000 U ml^−1^; Gibco) at 37°C in an 95% air and 5% CO_2_ gas mixture. SeroTabs were incubated in the cell culture medium at a medium-to-sample area ratio of 1.25 ml cm^−2^ for 24 hours at 37°C in a humidified 5% CO_2_ atmosphere to obtain extracts, which potentially contain cytotoxic components such as magnetic particles or degradation byproducts of the polymer matrix. These extracts were subsequently collected and used for cytotoxicity evaluation. The viability of HT29 cells were measured by the CellTiter-Glo (CTG) assays (Promega, Madison, WI). Briefly, HT29 cells were harvested and seeded in 96-well plates at a density of 9000 cells per well and preincubated for 24 hours. Then, the culture medium was replaced with the prepared extracts of SeroTabs and cultured for an additional 24, 48, and 72 hours. Following the incubation period, cell viability was measured using the standard CTG assay according to the manufacturer’s instructions (fig. S20).

To compare the cytocompatibility of PrFeB and SmFeN magnetic particles, Normal Human Dermal Fibroblasts (NHDF) cells were cultured in DMEM (Gibco, catalog no. 11965092), supplemented with 10% FBS (Gibco, catalog no. 10270106) and 1% penicillin-streptomycin (Gibco, catalog no. 15140122). Cells were maintained at 37°C in a humidified 5% CO_2_ incubator and passaged every 2 to 3 days using 0.05% trypsin-EDTA (Gibco; catalog no. 25300062). Cell viability was evaluated using the CCK-8 assay. NHDF cells were seeded in 96-well plates (Corning, catalog no. 3596) at 1 × 10^4^ cells per well in 100 μl of complete culture medium and allowed to attach for 24 hours. After attachment, the medium was replaced with 100 μl of fresh medium containing magnetic particles at final concentrations of 0.1 to 10 mg/ml (corresponding to 0.01 to 1 mg per well). Cells were incubated with the particles for 24 hours (day 1) and 72 hours (day 3) at 37°C and 5% CO_2_. At each time point, the exposure medium was removed and replaced with 100 μl of culture medium containing 10% CCK-8 reagent (Abcam, catalog no. ab228554) followed by 1 hour of incubation. Absorbance was measured at 450/650 nm using an Infinite F200 PRO microplate reader (Tecan).

We performed a dose-response viability assay using concentrations ranging from 0.01 to 1 mg per well and assessed metabolic activity on days 1 and 3 (fig. S22). PrFeB particles exhibit a clear, dose-dependent reduction in viability, remaining above 90% only at concentrations ≤0.05 mg per well. At 0.1 mg per well, viability decreases to ~80%, indicating the onset of cytotoxic effects, whereas at higher doses (0.5 to 1 mg per well) metabolic activity drop sharply to 38 to 47%, demonstrating marked toxicity at elevated concentrations. In contrast, SmFeN particles show substantially higher cytocompatibility, maintaining >100% viability at low doses (0.01 to 0.05 mg per well) and remaining nontoxic up to 0.5 mg per well. Even at 1 mg per well, SmFeN preserves >80% viability, indicating that only the highest concentrations approach cytotoxic levels, whereas lower doses remain safe. Collectively, these findings demonstrate that both particle types are biocompatible within their low-dose working ranges, but SmFeN exhibits a substantially broader safety window and becomes cytotoxic only at the uppermost concentrations tested.

### Evaluation of biocompatibility

For histological analysis, the esophagus and stomach tissues from each rabbit group were collected after surgery and immersed in 4% formaldehyde for H&E staining. Tissue was aseptically dissected and fixed overnight at 4°C in 10% formalin. Subsequently, the samples were dehydrated through a graded ethanol series from 70 to 100%, cleared with xylene to remove the ethanol, and infiltrated with paraffin. The processed tissue was embedded in paraffin, sectioned into 5-μm-thick slices, placed on microscope slides, and stained with H&E for histological examination. Images of stained tissue sections were acquired using a light microscope with a slide scanner (Pannoramic 250/MIDI, 3D HISTECH Ltd., Hungary).

## Supplementary Materials

### The PDF file includes:

Supplementary Text Figs. S1 to S23 Legends for movies S1 to S11 References
