# Supplementary material for: Electronics-free soft robotic minitablet for on-demand gastric molecular sensing and diagnostics in vivo
Source: Sci Adv. 2026 May 8;12(19):eaea3309. doi: 10.1126/sciadv.aea3309 (PMC13155308; doi:10.1126/sciadv.aea3309)
Supplement: Supplementary file 1 — Supplementary Text Figs. S1 to S23 Legends for movies S1 to S11 References [file sciadv.aea3309_sm.pdf]

Science Advances
AAAS

Supplementary Materials for

Electronics-free soft robotic minitablet for on-demand gastric molecular sensing and diagnostics in vivo

Chen Wang *et al.*

Corresponding author: Xiang-zhong Chen, xzchen@fudan.edu.cn; Zongwei Cai, zwcai@hkbu.edu.hk; Sarthak Misra, s.misra@utwente.nl; Venkatasubramanian K. Venkiteswaran, v.kalpathyvenkiteswaran@utwente.nl

Sci. Adv. **12**, eaea3309 (2026)
DOI: 10.1126/sciadv.aea3309

The PDF file includes:

Supplementary Text
Figs. S1 to S23
Legends for movies S1 to S11
References

Other Supplementary Material for this manuscript includes the following:

Movies S1 to S11

Supplementary Text

Analysis of the magnetic actuation force

To evaluate the mechanical response of SeroTab under predefined magnetization profiles, we performed finite element analysis using COMSOL Multiphysics to compute the magnetic field distribution of the permanent magnets and the resulting magnetic forces acting on the device. The locomotion of SeroTab was driven primarily by magnetic pulling forces generated by the spatial gradient of the field, which directs movement from regions of lower to higher magnetic flux density. To exploit this effect, we utilized the edge region of a cylindrical permanent magnet, where the field was both strong and highly non-uniform. Additionally, the programmed magnetization profile of SeroTab allowed for controllable orientation under external magnetic fields, enabling both directional alignment and bending deformation. We divided the magnetic force (**F**) acting on SeroTab into three components: the magnetic pulling force (F_x), which drives forward sliding locomotion; the magnetic rotating force (F_y), which rotates the SeroTab; and the magnetic lifting force (F_z), which counteracts gravity and facilitates flipping. The Magnetic force (**F**) and magnetic torque (**M**) acting on SeroTab are defined by the following equations:

$$\mathbf{F} = \nabla((\iiint_{V_{Mag}} \mathbf{m} dV_{Mag}) \cdot \mathbf{B}) \tag{S1}$$

$$\mathbf{M} = \iiint_{V_{\text{Mag}}} \mathbf{m} \times \mathbf{B} \, dV \tag{S2}$$

where $\mathbf{B} = (B_x, B_y, B_z)$ is the magnetic flux density generated by the permanent magnet at point (x, y, z), $\mathbf{m} = (m_x, m_y, m_z)$ is the magnetization of SeroTab (see (Fig. S6)), V_{Mag} represents the volume of MPC within SeroTab.

We investigated the magnetic actuation performance of single-, dual-, and four-magnet configurations to optimize the applied magnetic forces (Fig. S4). Magnetic forces were calculated by sweeping three key parameters: the magnet's rotation angle (θ), the horizontal distance (d), and the vertical distance (h) between the magnet and SeroTab. In the single-magnet setup, the magnetic pulling force peaked when the magnet was rotated to 90°. Introducing a second magnet on the opposite side significantly enhanced the pulling force while reducing the lifting component, compared to placing both magnets on the same side. The four-magnet configuration yielded the highest

overall pulling force; however, the increase was not linear and does not result in a doubling of force. In summary, the single-magnet configuration offers ease of operation and greater flexibility in orientation, while the dual-magnet (opposing) setup effectively strengthens the pulling force and minimizes the undesired lifting force.

Based on the theoretical results, we constructed both single- and dual-magnet systems to experimentally validate the magnetic actuation of SeroTab. The magnetic field was measured using a Gaussmeter, and the magnetic force was quantified using a custom-designed two-axis force sensing platform (Fig. S5). The experimental data showed strong agreement with the simulation results, confirming the accuracy of the model. Additionally, the measurements revealed the optimal spatial relationship between SeroTab and the magnet for maximizing the pulling force.

Analysis of ultrasonic pH sensing

The ultrasound images were acquired using a medical ultrasound system (EPIQ7c, Philips, Netherlands) equipped with a C1-5 broad-spectrum convex array transducer (Philips). To ensure image clarity, ultrasound gel was applied at the probe-skin interface, and imaging parameters including gain, dynamic range, frequency, and focal depth were manually adjusted to align with the location of SeroTab. High-quality B-mode images were obtained, in which the embedded Zn disks appeared as distinct bright segments. The measurement tool within the ultrasound system was used to determine the distance between the left and right Zn markers, enabling quantification of pH-responsive deformation.

Selection of metal (Zn) as ultrasonic indicators took into consideration their biocompatibility and acoustic mismatch with the surrounding hydrogel, silicone, and soft tissues (*41, 58*). To enhance the ultrasound visibility of the embedded Zn markers in SeroTab, their dimensions were optimized based on acoustic impedance mismatch theory. The acoustic impedance of a material is defined as:

$$Z = \rho c \tag{S3}$$

where Z is the acoustic impedance, ρ is the material density, and c is the speed of sound in the medium.

At the interface between two materials, the reflection of ultrasound waves is governed by the

reflection coefficient R, calculated as:

$$R = \left|\frac{Z_2 - Z_1}{Z_2 + Z_1}\right|^2 \tag{S4}$$

where Z_1 and Z_2 are the acoustic impedances of the two interfacing media. A greater mismatch leads to a higher reflection coefficient and thus stronger echogenic signals in B-mode ultrasound imaging.

Zinc (Zn) exhibits a high acoustic impedance of approximately 26.7 MRayl, which contrasts strongly with surrounding materials such as hydrogel (~1.5 MRayl), gastric fluid (~1.6 MRayl), and silicone rubber (~1.0 MRayl). This results in a reflection coefficient $R > 0.85$, sufficient for generating bright and distinct echoes in ultrasound images.

To determine the appropriate thickness of the Zn disk, we consider the ultrasound wavelength λ in the surrounding medium:

$$\lambda = \frac{c}{f} \tag{S5}$$

where f is the operating frequency of the transducer. Given an ultrasound frequency of 5 MHz and average sound speed in tissue ($c \approx 1500$ m/s), the corresponding wavelength is $\lambda \approx 300$ μm. The Zn disk thickness is chosen as 50 μm, corresponding to approximately $\lambda/6$, which is sufficient to generate detectable reflection while minimizing acoustic shadowing or harmonic distortion.

Zn markers were fabricated with a diameter of 1 mm and thickness of 50 μm. This size provided a localized, high-contrast signal without excessive interference with surrounding structures. Marker spacing and alignment were optimized to remain within the axial and lateral resolution limits of the ultrasound system, ensuring consistent visibility during *in vivo* imaging (*41*).

Analysis of liquid sampling procedure

Liquid absorption relies on the pressure differential between the internal chamber and the external environment, which is generated by the elastic recovery of the shape memory polymer (SMP) actuator. Upon thermal activation, the SMP recovers its original shape, leading to an increase in the chamber volume and a corresponding decrease in internal pressure. This pressure difference between the surrounding environment and the chamber drives the intake of fluid through the microchannel, analogous to the working principle of an eyedropper. Based on this mechanism, we

conducted a simulation of the fluid sampling process using COMSOL Multiphysics to evaluate the dynamic pressure response and fluid inflow behavior.

Assuming isothermal conditions and negligible gas exchange with the environment, the internal pressure $P(t)$ of the chamber at time t can be described using a simplified inverse relationship:

$$P(t) = P_0 - \frac{1}{k}V(t) \tag{S6}$$

where P_0 is the atmospheric pressure, $V(t)$ is the chamber volume at time t (Fig. S10), k is a system-dependent constant that incorporates chamber geometry and gas properties.

As the volume $V(t)$ increases due to SMP deformation, the internal pressure $P(t)$ decreases, generating a pressure gradient $\Delta P = P_0 - P(t)$ across the microchannel. This gradient induces liquid inflow without the need for external pumps. The absorbed fluid volume is governed by the magnitude of the pressure difference (ΔP), the viscosity and surface tension of the surrounding fluid, as well as the contact angle between the fluid and the microchannel surface. In addition, the effect of capillarity is analyzed to minimize unintended passive fluid ingress and optimize the reliability of controlled sampling (*64, 65*). According to the Lucas–Washburn (LW) equation (*66*), which describes capillary-driven flow as:

$$\frac{dh}{dt} = \frac{(P_A + P_h + P_c)(r^2 + 4\varepsilon r)}{8\mu h} \tag{S7}$$

where h is the liquid column height, r is the channel radius, μ is the liquid viscosity, ε is the slip coefficient, P_A is the unbalanced atmospheric pressure, P_h is the hydrostatic pressure, and P_c is the capillary pressure. The capillary pressure is defined as:

$$P_c = \frac{2\sigma \cos\theta}{r} \tag{S8}$$

where σ is the liquid surface tension and θ is the contact angle at the channel wall.

According to the model, capillary-driven flow was strongly influenced by the channel radius, surface tension, contact angle, and fluid viscosity. To accurately simulate the fluid behavior within the SeroTab, we experimentally measureed the surface tension and contact angle of gastric juice extracted from porcine stomachs (Fig. S15 and Fig. S16). Based on these measured properties, we conducted time-dependent capillary flow simulations in COMSOL Multiphysics (Fig. S14B). The

simulation results indicated that the capillary effect within the SeroTab's microchannel is minimal under the tested conditions, primarily due to the inherent properties of the native materials and the specialized design of the microchannel structure.

Design and optimization of the Tesla valve

To enhance flow rectification and minimize unintended liquid leakage during operation, a Tesla-type (T45-R) microvalve was integrated into SeroTab's microfluidic channel (Fig. S12A). The design aimed to maximize the diodicity (D_i), defined as the ratio of the pressure drop in the reverse flow direction ($\Delta p_{\leftarrow}$) to that in the forward flow direction ($\Delta p_{\rightarrow}$) (*67, 68*):

$$D_i = \frac{\Delta p_{\leftarrow}}{\Delta p_{\rightarrow}} \tag{S9}$$

A higher diodicity value reflects stronger resistance to reverse flow, ensuring robust directional fluid transport during the sampling process.

The Tesla valve geometry is parameterized by key structural variables, including the curvature radius (R), turning angles (α, β), and segment lengths (L_1, L_2, L_3, L_4), as illustrated in Fig. S12A. The segment lengths L_1 and L_2 are calculated based on geometric relationships as:

$$L_1 = R\sin\beta + R\cos\left(\frac{\pi}{2} - \theta\right) + \frac{R\cos\beta + R\sin\left(\frac{\pi}{2} - \theta\right)}{\tan\theta} \tag{S10}$$

$$L_2 = \sqrt{\left(R\sin\left(\frac{\pi}{2} - \theta\right) + R\cos\beta\right)^2 + \left(\frac{R\cos\beta + R\sin\left(\frac{\pi}{2} - \theta\right)}{\tan\theta}\right)^2} \tag{S11}$$

where θ is the turning angle within the Tesla structure.

Considering the inertial-dominated regime where Tesla valves operate effectively (typically at moderate Reynolds numbers, $Re \sim$ 10–1000), finite element simulations are conducted in COMSOL Multiphysics to optimize the geometric parameters. The final design achieved optimal performance with $R = 1.1$ mm, $\alpha = 41°$, and $\beta = 45°$ (Fig. S12A).

Flow field simulations (Fig. S12B) showed significant asymmetry between forward and reverse directions, and the flow diodicity increases markedly with increasing Reynolds number (Fig. S12C). This optimized Tesla microvalve structure effectively restricts backflow, stabilizes fluid sampling, and minimizes unintended liquid leakage within the SeroTab system.

To evaluate the functional contribution of the Tesla valve in SeroTab, we performed comparative impact-induced leakage tests using devices with and without the Tesla valve. A control device was fabricated by replacing the Tesla valve with a straight microchannel of identical cross-section and material, and using the same fabrication process (Fig. S13A). Each device was pre-filled with a fixed volume of dyed buffer and subjected to different mechanical disturbances, including a 1 m free fall and the release of 10 g and 50 g weights from a 20 cm height. Leakage was quantified gravimetrically, and representative visual evidence is shown in Fig. S13B and Movie S11. Each impact condition was repeated five times to ensure statistical reliability. Across all impact conditions, the Tesla-valve device exhibited effective leakage prevention, whereas the straight-channel control showed substantially greater leakage. The results confirm that, although the Tesla valve does not function as a complete check valve under static or low-flow conditions, it effectively suppresses reverse flow during transient mechanical shocks, thereby minimizing unintended loss of the collected gastric fluid.

From a broader perspective, the Tesla valve in its current passive form provides a lightweight and fabrication-compatible strategy to suppress impact-induced backflow. However, integrating more sophisticated micro-scale check-valve architectures in future designs may further enhance post-sampling contamination prevention and enable complete blockage of reverse flow even under static pressure conditions. Achieving this will require continued innovation in microfabrication techniques and the incorporation of smart or responsive materials capable of active sealing.

Analysis of inductive heating procedure

Remote heating can be achieved using alternating magnetic fields or radio-frequency (RF) fields through two primary mechanisms: Joule heating and hysteresis losses. While magnetic hysteresis is widely used for remote heating due to its compatibility with multifunctional nanoparticles, Joule heating offers higher efficiency and greater controllability, making it a more effective approach for precise thermal management (*52*). The selection and design of the heating element were based on the fundamental principles of electromagnetic induction and transient thermal energy balance. The heating power generated within the material under an alternating magnetic field primarily results from eddy currents, and the volumetric heating power density P is given by:

$$P = \frac{J^2}{\sigma} \tag{S12}$$

where J is the induced current density and σ is the electrical conductivity of the material. To maximize heating efficiency, a material with high electrical conductivity and low magnetic susceptibility was preferred. Aluminum was selected over other candidates such as iron and copper due to its high conductivity ($\sigma_{\text{Al}} \sim 3.5 \times 10^7$ S/m), low density (2.7 g/cm^3), biocompatibility, and the absence of magnetic hysteresis losses which would otherwise complicate heating control.

The geometry of the aluminum film was optimized to enhance heating performance while maintaining flexibility and minimizing energy requirements. The characteristic skin depth δ, which defines the penetration depth of the induced currents, is given by:

$$\delta = \sqrt{\frac{2}{\omega \mu \sigma}} \tag{S13}$$

where $\omega = 2\pi f$ is the angular frequency, μ is the magnetic permeability, and σ is the electrical conductivity.

We used a 10 μm-thick aluminum film which was cut into the same shape as SMP to transfer the heating and ensure flexibility. To further enhance localized heating without sacrificing the global flexibility of the structure, an additional 100 μm-thick aluminum circular disk was laminated at the center of the thin film. This layered configuration increases local thermal energy generation where needed, while keeping the overall thermal mass low.

The heat generated within the aluminum sheet follows the transient energy conservation equation:

$$P_{\text{in}} = \rho V c_p \frac{\partial T}{\partial t} + H_L \tag{S14}$$

where ρ is the density, V is the volume of the aluminum, c_p is the specific heat capacity, $\partial T / \partial t$ is the temporal temperature rise rate, and H_L represents the heat loss to the surroundings.

Heat losses occur through convection and radiation, which are modeled as:

$$H_L = hA(T_s - T_{\text{ambient}}) + \sigma \epsilon A(T_s^4 - T_{\text{ambient}}^4) \tag{S15}$$

where h is the convective heat transfer coefficient, A is the exposed surface area, ϵ is the surface emissivity, and σ is the Stefan–Boltzmann constant. Minimizing H_L is essential to achieve rapid heating; therefore, the device is designed with compact geometry and encapsulated with low-conductivity materials to reduce convective and radiative losses.

Furthermore, the placement of the external RF coil relative to the SeroTab significantly influences the induced magnetic flux density B, which decreases with distance approximately as:

$$B(r) \propto \frac{1}{r^3} \tag{S16}$$

where r is the separation distance. A coil-to-device distance of 10–20 mm is selected to balance strong magnetic coupling with safe operation within biological tissues.

We conducted multivariate experiments to investigate the heating efficiency influenced by various factors, including the power of the RF heater, heating time, operating distance, and material thickness. The results, presented in Fig. S8, suggest that heating efficiency can be improved by using a higher power heater and a thicker aluminum disk, which together enhance the ability to heat over larger distances, making them suitable for larger animal experiments. In conclusion, careful selection of aluminum, optimization of thickness and layered structure, precise control of external coil positioning, and reduction of thermal losses collectively contribute to efficient, rapid, and localized heating of the actuator, achieving the required transition temperature (40 °C) within approximately 60 seconds under *in vivo* conditions.

To evaluate the heating robustness of the SMP actuation system, we performed a controlled benchtop experiment in which the aluminum disk was subjected to fixed RF heating parameters (60 s heating duration, 450 W RF power, a 2 cm heating distance, and a 100 μm aluminum disk thickness). During the test, the aluminum disk was incrementally rotated relative to the RF coil in 2° steps, and the surface temperature was recorded every 10 s over a 60 s activation period. The results show that the SMP reached its activation threshold of 40 °C within 30 s for angular offsets up to 6°, indicating that minor orientation variations do not meaningfully compromise heating efficiency (Fig. S9). Furthermore, as shown in Fig. S8, heating performance can be additionally compensated by adjusting operational parameters such as heating duration, RF power, distance, and aluminum thickness. Collectively, these findings demonstrate that the system maintains reliable and flexible thermal performance under physiologically relevant orientation variations in the dynamic

gastrointestinal environment.

Ultrasound measurement and resolution

The quality of ultrasound imaging is primarily determined by the lateral resolution and the signal-to-noise ratio (SNR), which together set the minimum detectable pH-induced dimensional change in the Zn disks. The lateral resolution is governed by the beamwidth, which depends on the wavelength, aperture size, and focal depth, as expressed in Eq. (S17). Higher transmit frequencies and stronger focusing result in narrower beams and improved resolution, whereas deeper focal positions broaden the beam and degrade spatial precision. The SNR is influenced by frequency-dependent acoustic attenuation, focusing conditions, and aperture size, with the highest SNR observed near the focal region and progressively reduced at greater imaging depths due to tissue scattering and attenuation.

$$\Delta x_{\mathrm{lat}} \propto \frac{\lambda F}{D} \tag{S17}$$

where λ is the ultrasound wavelength, F is the focal depth, and D is the aperture length of the transducer.

To analytically estimate the minimum detectable change in lateral spacing between two point-like scatterers, we model the lateral point spread function (PSF) of the ultrasound imaging system as a Gaussian function, a widely used approximation for phased arrays and convex probes in the focal region:

$$g(x) = A \exp\left(-\frac{x^2}{2\sigma^2}\right) \tag{S18}$$

where A is the peak amplitude and σ is the standard deviation of the Gaussian. The full width at half maximum (FWHM), corresponding to the lateral resolution of the system, is related to σ by:

$$\mathrm{FWHM} = 2\sqrt{2\ln 2}\,\sigma \approx 2.355\sigma \tag{S19}$$

For two point-like targets separated by distance d, the valley intensity between them is:

$$I_v(d) = 2A \exp\left(-\frac{d^2}{8\sigma^2}\right) \tag{S20}$$

A small change in spacing Δd produces a corresponding change in valley intensity, approximated using a first-order Taylor expansion:

$$|\Delta I_v| \approx \left|\frac{\partial I_v}{\partial d}\right| \Delta d = \frac{Ad}{2\sigma^2} \exp\left(-\frac{d^2}{8\sigma^2}\right) \Delta d \tag{S21}$$

To be detectable against background noise with standard deviation σ_n, this intensity change must exceed a threshold $k\sigma_n$ ($k \approx 1$–3):

$$|\Delta I_v| \gtrsim k\sigma_n \tag{S22}$$

Solving for the smallest detectable spacing change yields:

$$\Delta d_{\min} \approx \frac{2\sigma^2}{d} \exp\left(\frac{d^2}{8\sigma^2}\right) \frac{k}{\mathrm{SNR}} \tag{S23}$$

where the image-domain signal-to-noise ratio is defined as:

$$\mathrm{SNR} = \frac{A}{\sigma_n} \tag{S24}$$

We performed numerical simulations to quantify how ultrasound parameters and target geometry influence the accuracy of lateral distance measurements. Using a baseline configuration of 5 MHz center frequency, 2 cm imaging depth, and Zn disks of 1 mm diameter and 50 μm thickness, we evaluat the relative distance error $\Delta d/d_0$. Increasing ultrasound frequency improves lateral resolution and reduces measurement error (Fig. S18A), whereas increasing imaging depth broadens the PSF and decreases SNR, leading to a monotonic increase in $\Delta d/d_0$ (Fig. S18B). Simulations incorporating variations in Zn disk diameter and thickness further show that larger or thicker reflectors yield higher echo amplitudes and improved noise robustness (Fig. S18C-D), thereby reducing measurement variability. These results collectively demonstrate how imaging settings and target geometry govern the minimum resolvable displacement in ultrasound-based localization.

Under the same conditions as the *in vivo* experiments (5 MHz, 2 cm depth, 1 mm × 50 μm Zn disks), the inter-disk spacing (which reflects the local pH value) was extracted from ultrasound images by analyzing the lateral intensity profile across the Zn-induced bright spots. As shown in Fig. S18E, a straight line passing through the three Zn disks is manually defined, and a one-dimensional gray-scale profile along this line is extracted using bilinear interpolation. The location

of each Zn disk is determined by identifying the point of maximum intensity gradient within the rising edge of its corresponding bright spot, corresponding to the onset of the acoustic reflection from the disk. The inter-disk spacing is then calculated as the pixel distance between these edge positions multiplied by the calibrated pixel-to-millimeter conversion factor obtained from the depth scale on the ultrasound image. Measurements from *in vivo* ultrasound frames (at 0 min and 60 min) consistently produced $\Delta d/d_0 < 0.1$, indicating that dimensional changes as small as 10% can be reliably detected under the experimental imaging conditions.

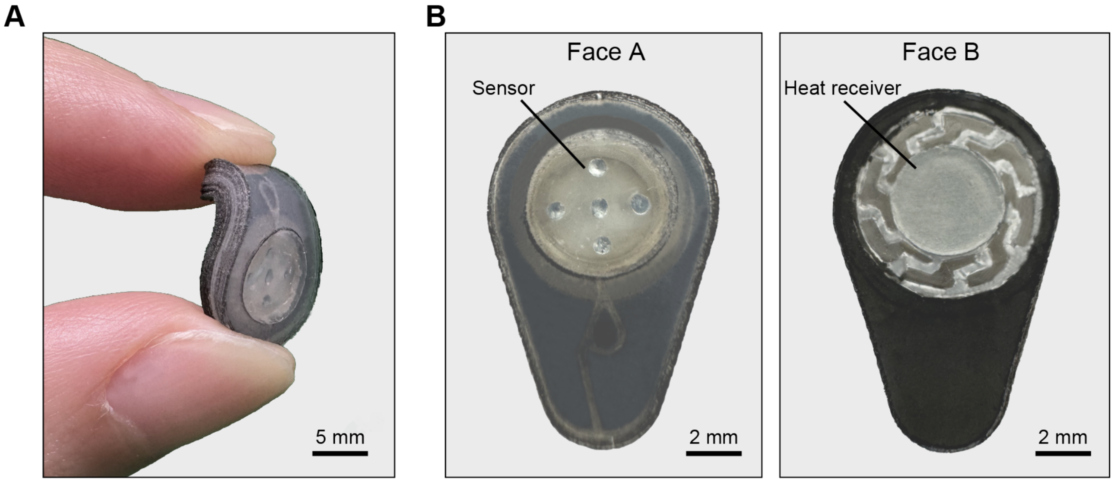

Figure S1: **Images of SeroTab that highlight the visual characteristics and soft features.** (**A**) SeroTab being compressed and bent between fingers, demonstrating its flexibility. (**B**) Views of both faces of the SeroTab, each highlighting different components.

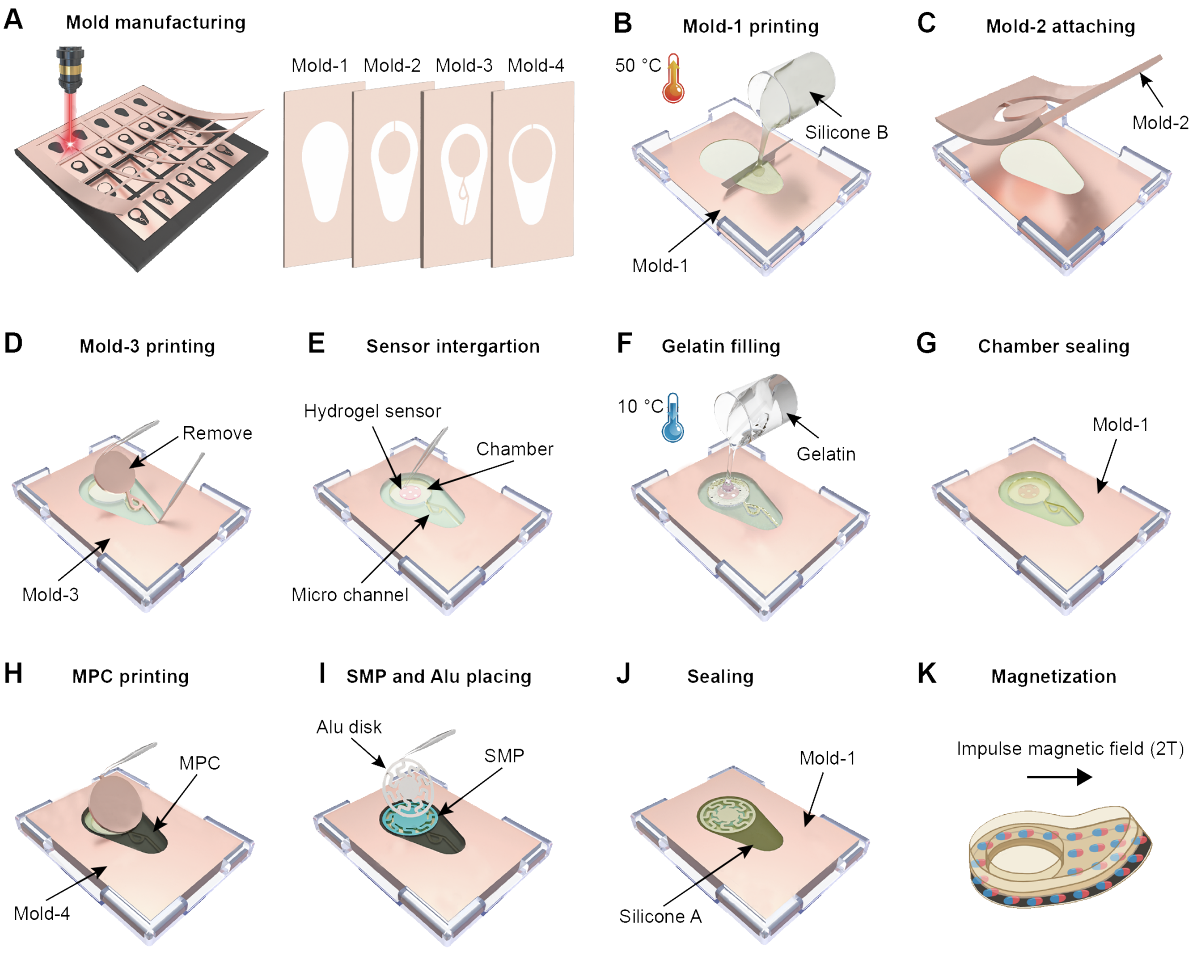

Figure S2: **Fabrication procedure of SeroTab.** (**A**) Laser cutting of four types of molds. (**B**) Printing of the SeroTab base using mold-1. (**C**) Printing of the circular chamber using mold-2. (**D**) Printing of the Tesla valve chamber using mold-3. (**E**) Placement of the hydrogel sensor and (**F**) gelatin filling for structural support. (**G**) Chamber sealing using mold-1. (**H**) Printing of the magnetic layer using mold-4. (**I**) Placement of the shape memory polymer (SMP) and aluminum disk. (**J**) Top sealing using mold-1. (**K**) Magnetization under a 2 T impulse magnetic field.

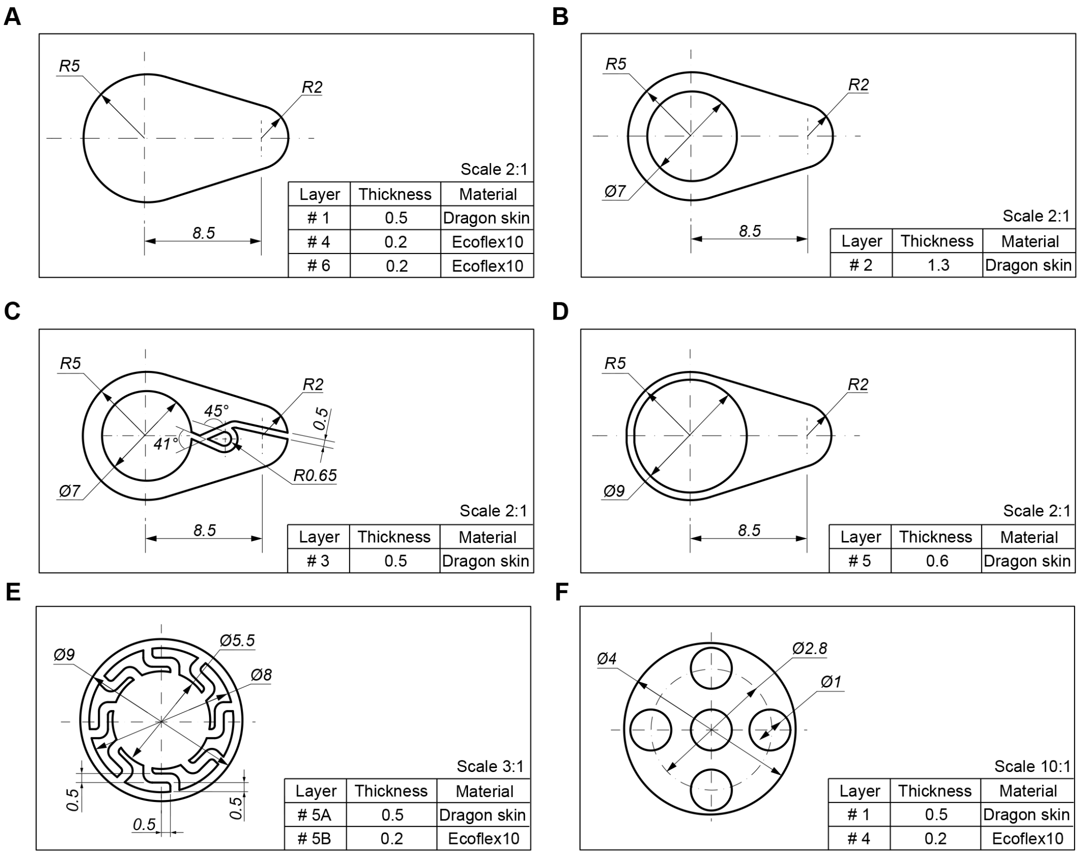

Figure S3: **Design and dimensions of different layers of SeroTab.** (**A**) Dimension and materials of layer 1, layer 4, layer 5, (**B**) layer 2, (**C**) layer 3, (**D**) layer 5, (**E**) SMP (layer 5A), aluminum heating element (layer 5B) and (**F**) pH sensor.

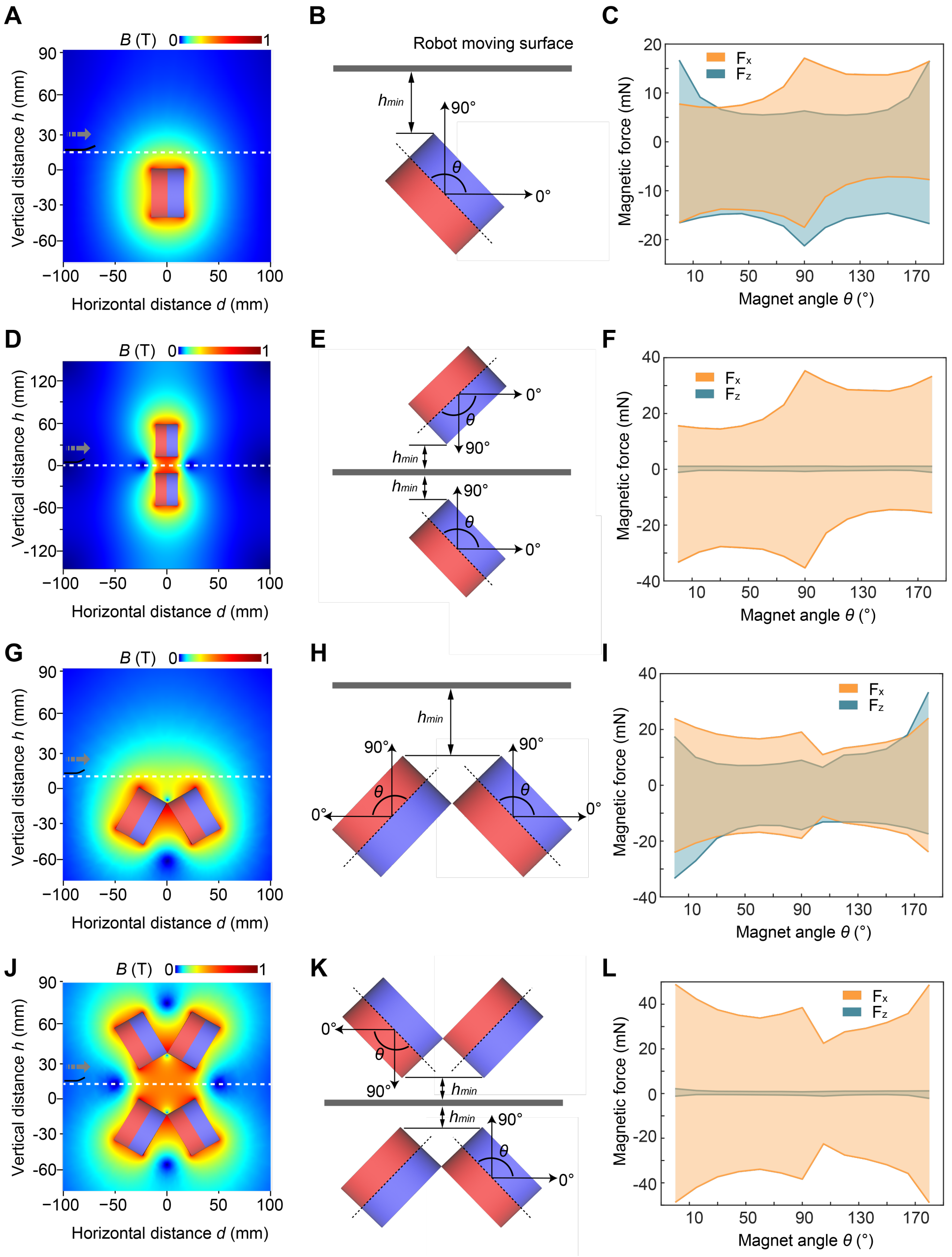

Figure S4: **Comparison and enhancement of applied magnetic force on SeroTab with different magnet configurations.** (**A**) Simulated magnetic field distribution map with a single magnet. (**B**) Schematic illustration of controllable variables influencing magnetic force. (**C**) Simulated results of pulling force (F_x) and lifting force (F_z) act on SeroTab. (**D**), (**E**) and (**F**) demonstrate the use of two magnets on opposite sides to enhance pulling force and reduce lifting force. (**G**), (**H**) and (**I**) show the effects of using two magnets on one side. (**J**), (**K**) and (**L**) illustrate the strategy of using four magnets to further optimize pulling force and minimize lifting force.

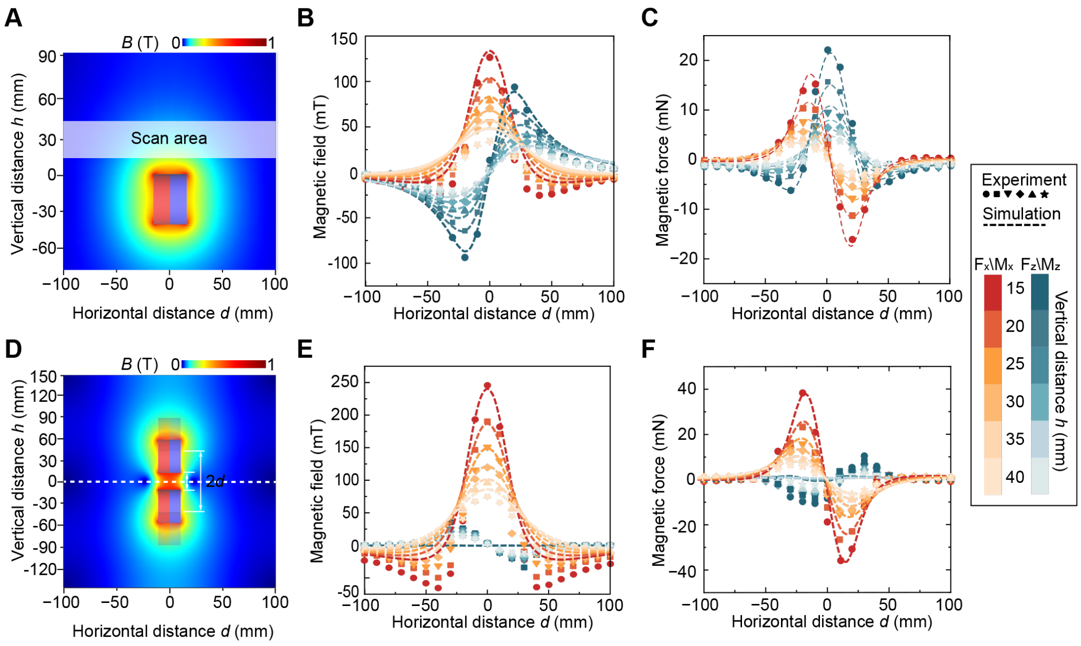

Figure S5: **Comparison of measured and simulated pulling force (F_x) and lifting force (F_z).** (**A**) Simulated magnetic field distribution map with a single magnet, showing the vertical and horizontal distance between SeroTab and the magnet. (**B**) Comparison of measured and simulated magnetic fields as a function of horizontal and vertical distance. (**C**) Comparison of measured and simulated magnetic forces influenced by horizontal and vertical distance. (**D**), (**E**) and (**F**) demonstrate the use of two magnets on opposite sides to enhance the pulling force and reduce lifting force.

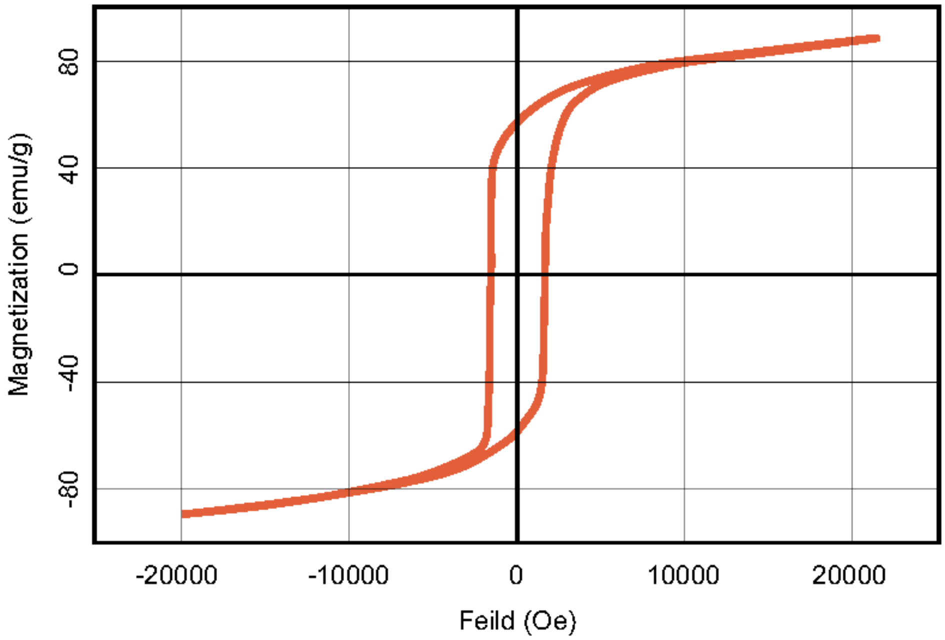

Figure S6: **Magnetization properties (B-H curve) of the magnetic polymer composite (MPC) in SeroTab.** The magnetization behavior of MPC is measured using a vibrating-sample magnetometer (VSM).

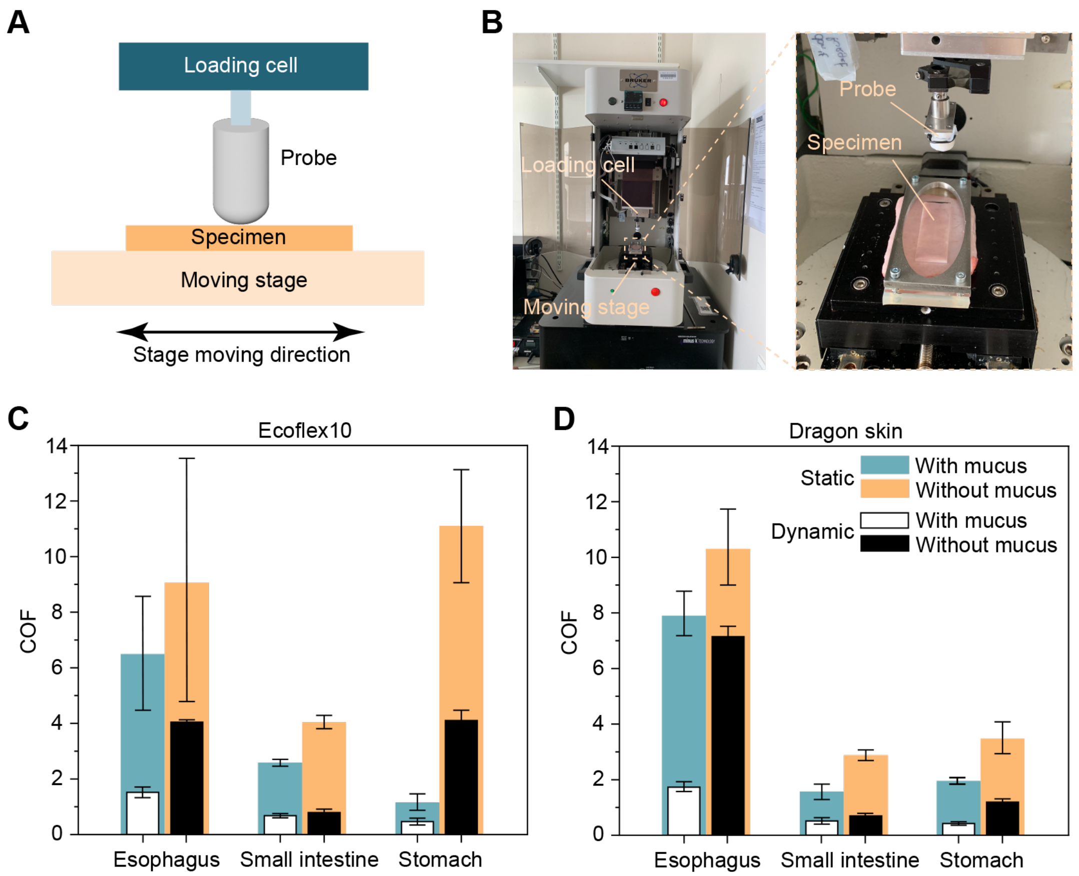

Figure S7: **Measurement of the coefficient of friction (COF) between SeroTab's materials and organ tissues.** (**A**) Schematic illustration of the COF measurement mechanism, where a piece of SeroTab's material is attached to the probe. (**B**) Experimental setup. (**C**) COF values for Ecoflex 10 and (**D**) Dragon skin in contact with tissues from the esophagus, small intestine, and stomach.

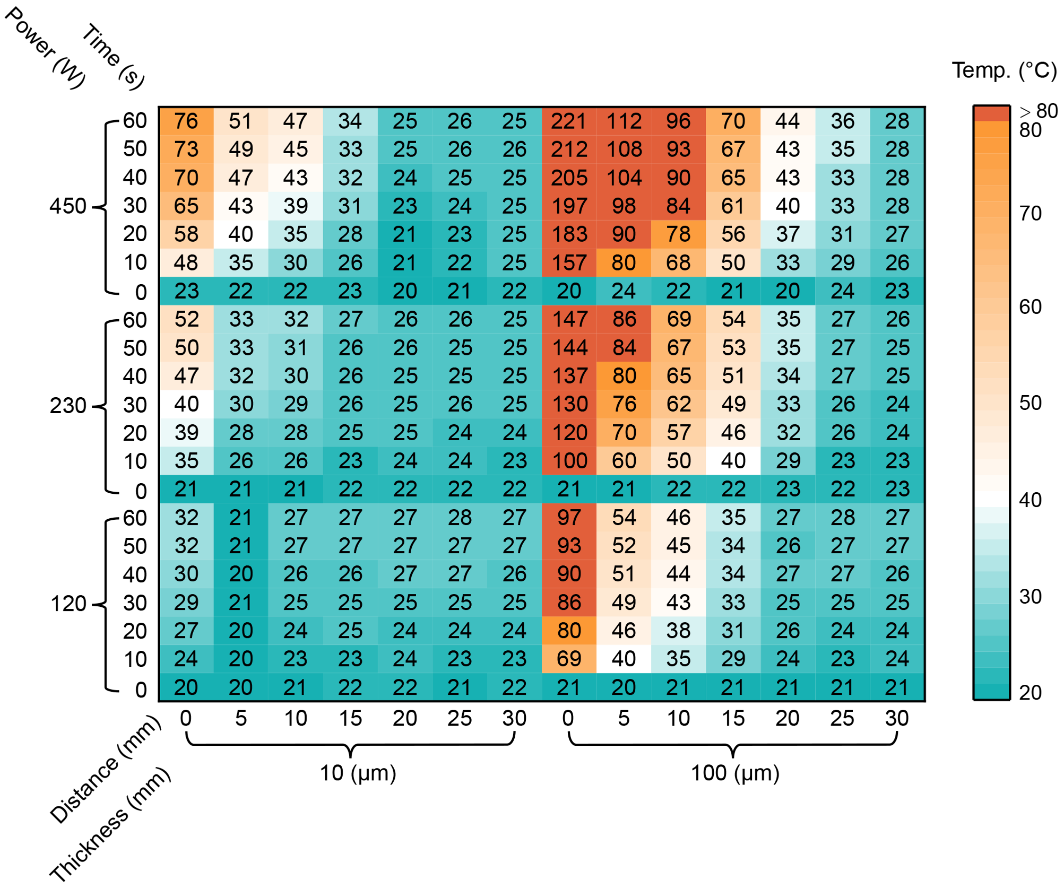

Figure S8: **Heating efficiency of the aluminum heating element.** A comprehensive data summary analyzing the effects of heating time, RF heater power, heating distance, and aluminum thickness on temperature.

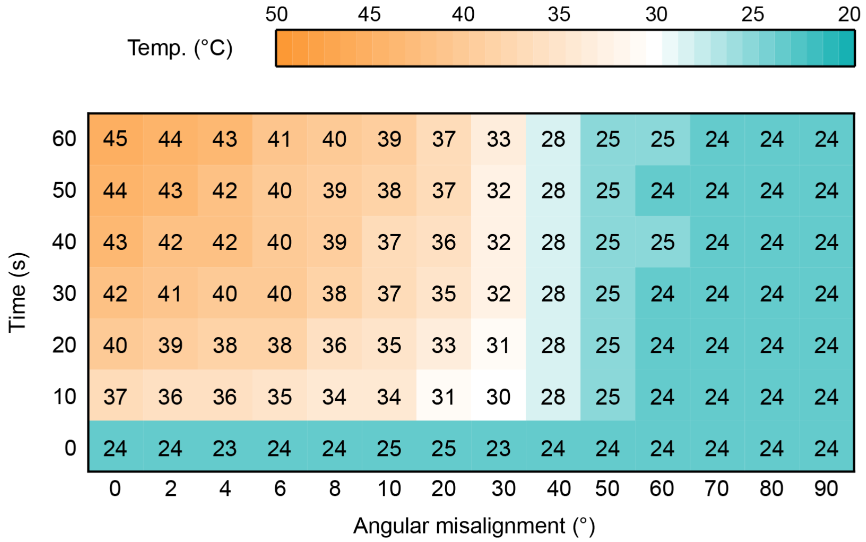

Figure S9: **Orientation effects on heating efficiency.** The heating time, RF heater power, heating distance, and aluminum thickness are set to be 60 s, 450 W, 2 cm, and 100 μm, respectively.

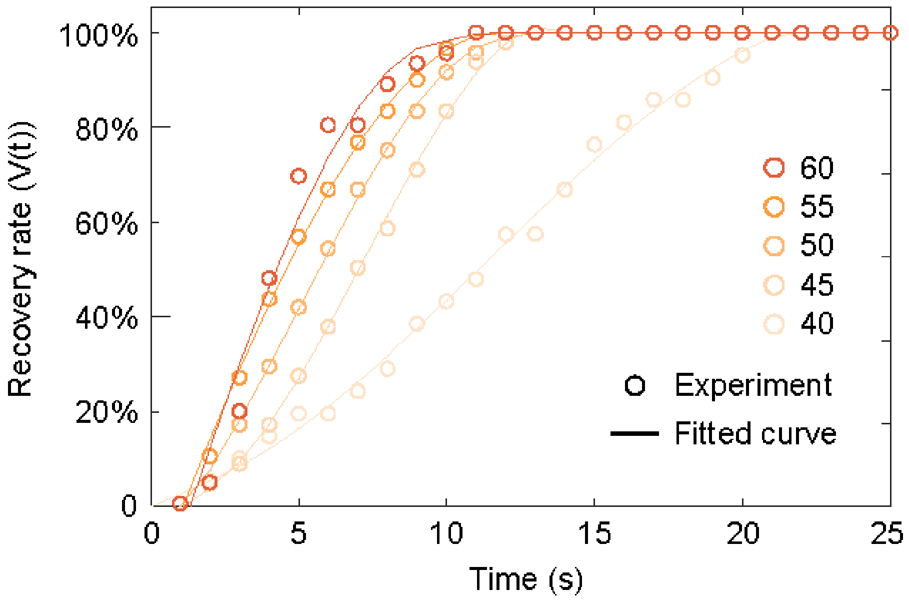

Figure S10: **Recovery rate of the SMP actuator at different temperatures.**

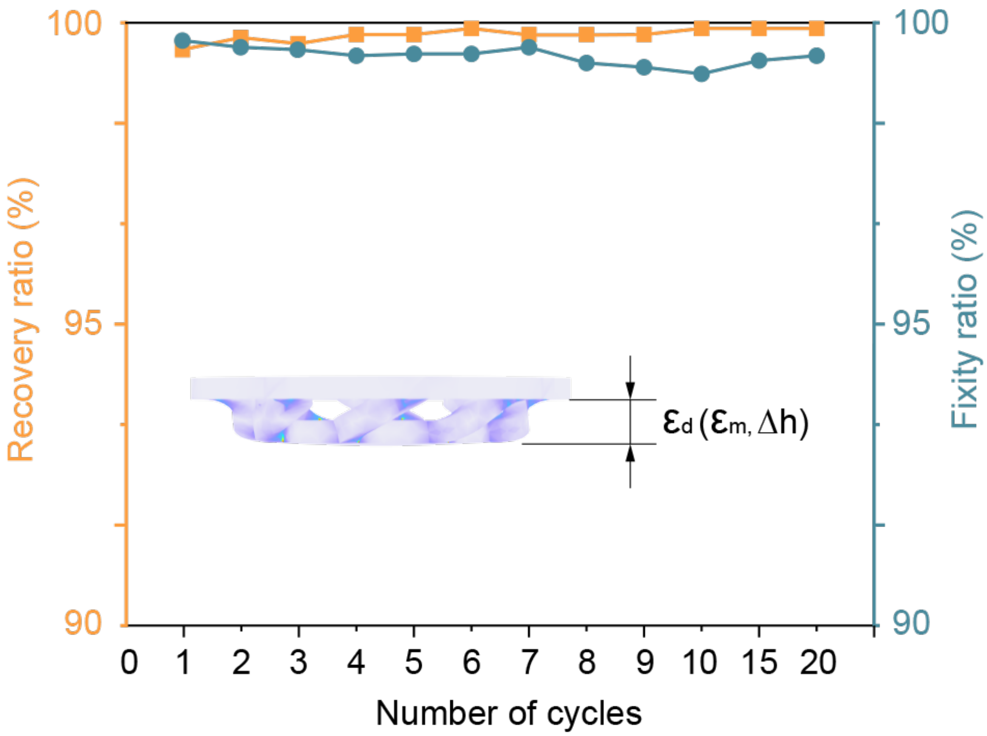

Figure S11: **Shape recovery ratio and fixity ratio of the SMP over repeated training cycles.**

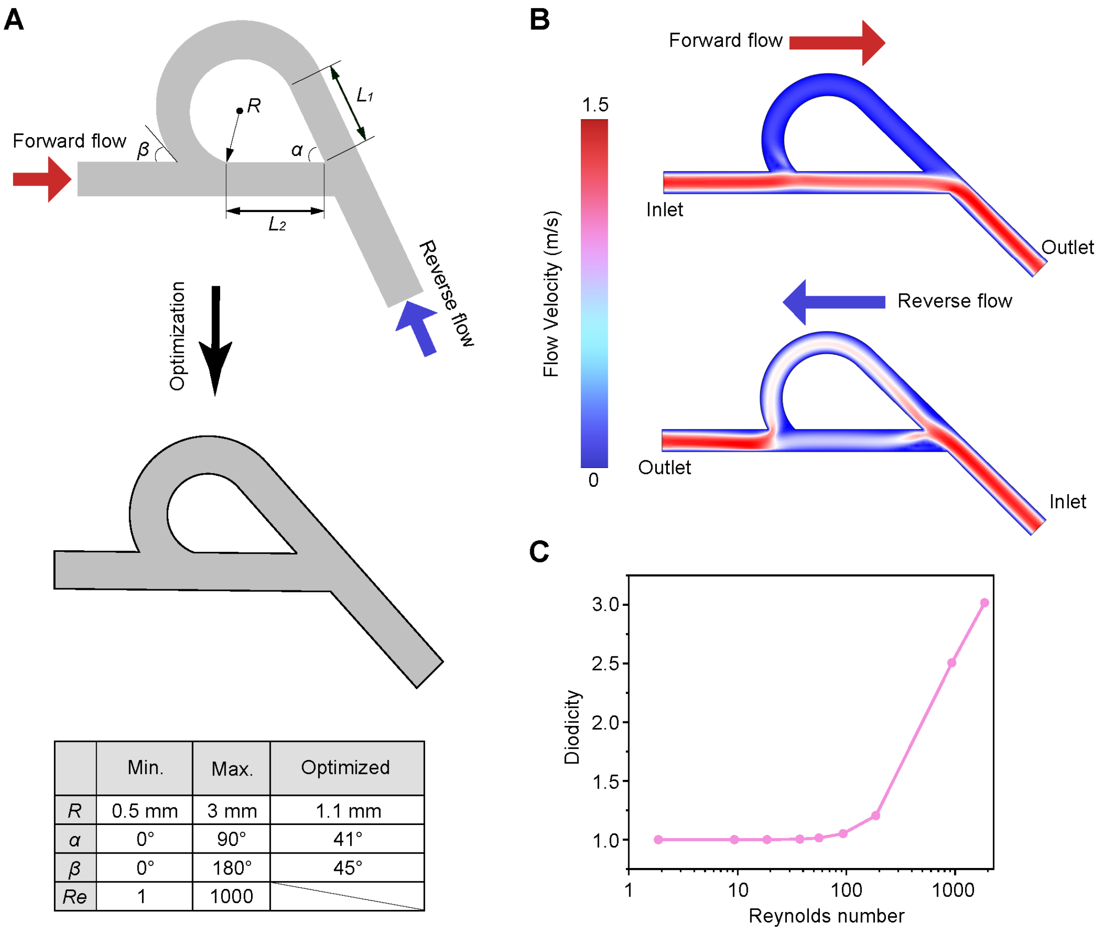

	Min.	Max.	Optimized
R	0.5 mm	3 mm	1.1 mm
α	0°	90°	41°
β	0°	180°	45°
Re	1	1000	

Figure S12: **Parameter optimization of the Tesla valve in SeroTab.** (**A**) Schematic illustration and table of optimized variables in the Tesla valve. (**B**) Simulated flow velocity of the Tesla valve in the forward direction (top) and reverse direction (bottom). (**C**) Simulated diodicity at different Reynolds numbers of the optimized Tesla valve.

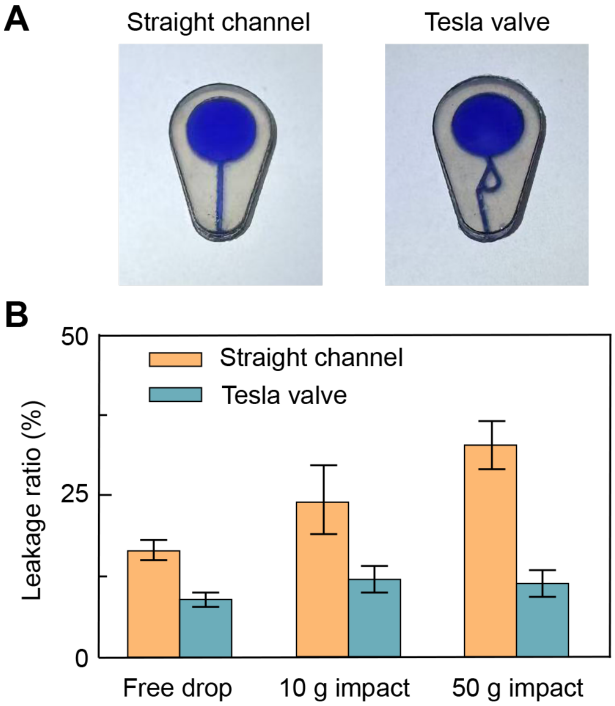

Figure S13: **Comparative evaluation of straight channels and Tesla valves for leakage minimization under impact loading.** (**A**) Images of the chamber–channel part with a straight channel and a Tesla valve, respectively. (**B**) Experimental results of leakage ratios under different impact conditions: free drop from 1 m, 10 g impact from 20 cm, and 50 g impact from 20 cm. Each impact condition is repeated five times to ensure statistical reliability.

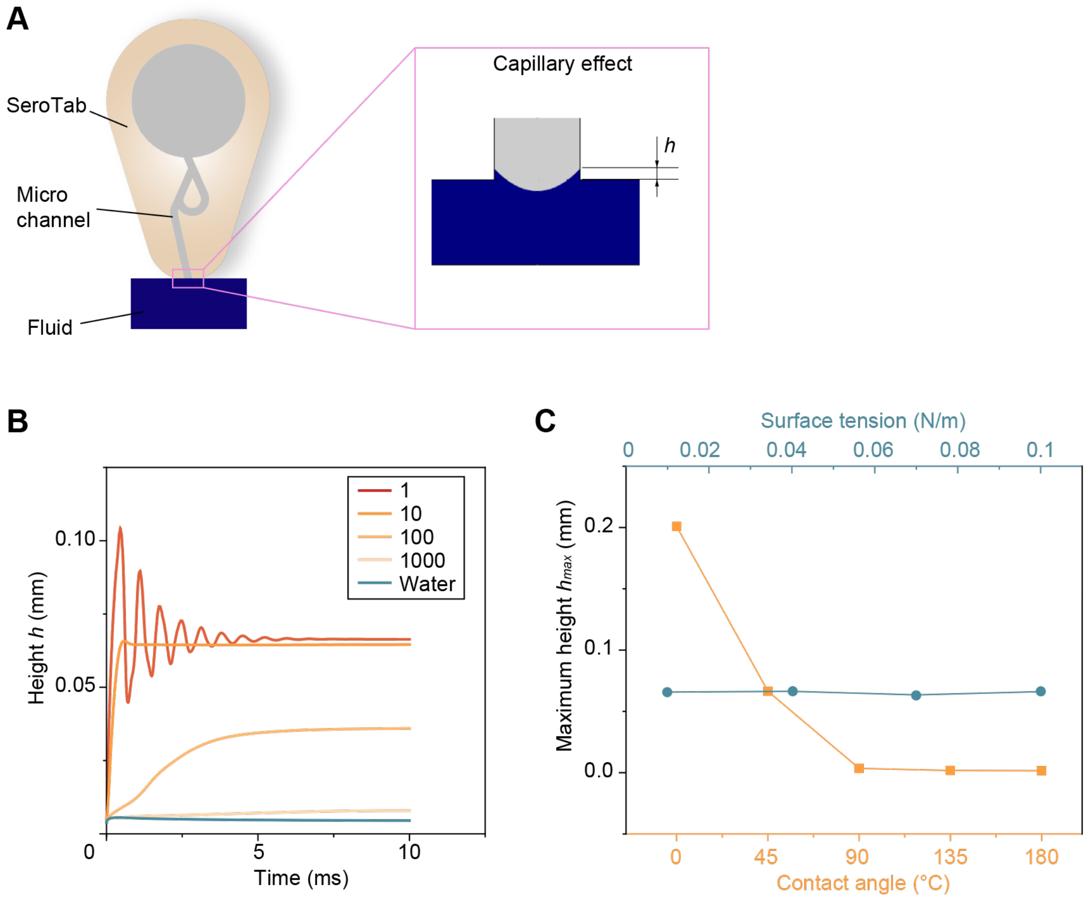

Figure S14: **Capillary effect in the microchannel of SeroTab.** (**A**) Simulated liquid level of the capillary effect using COMSOL Multiphysics 6.3. (**B**) Time-dependent liquid level height for liquids with different viscosities. (**C**) Simulation results illustrating the influence of liquid properties on the maximum liquid level height.

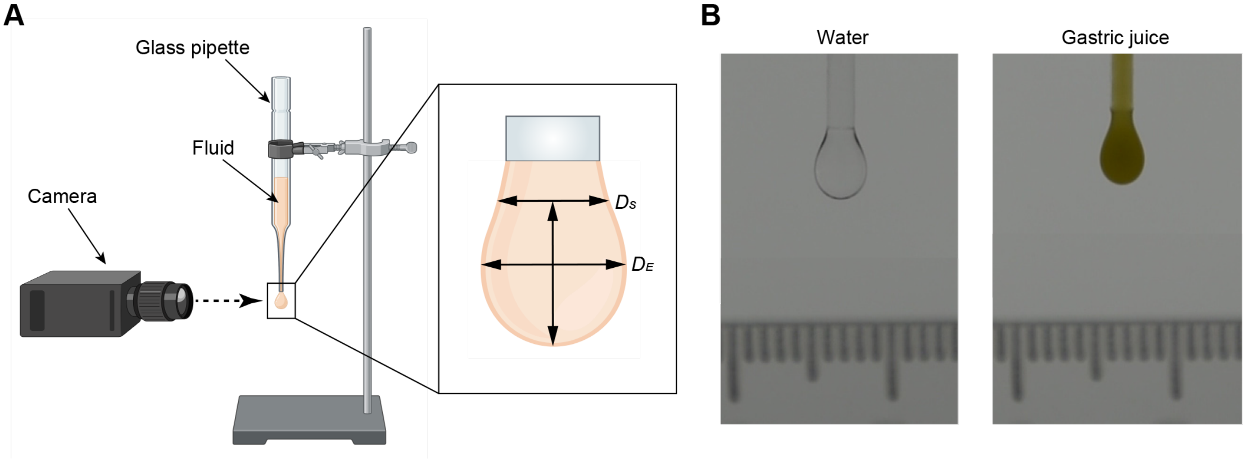

Figure S15: **Measurement of liquid surface tension** (**A**) Schematic illustration of the mechanism for measuring surface tension. (**B**) Images of water and gastric juice droplets captured for the measurement of surface tension.

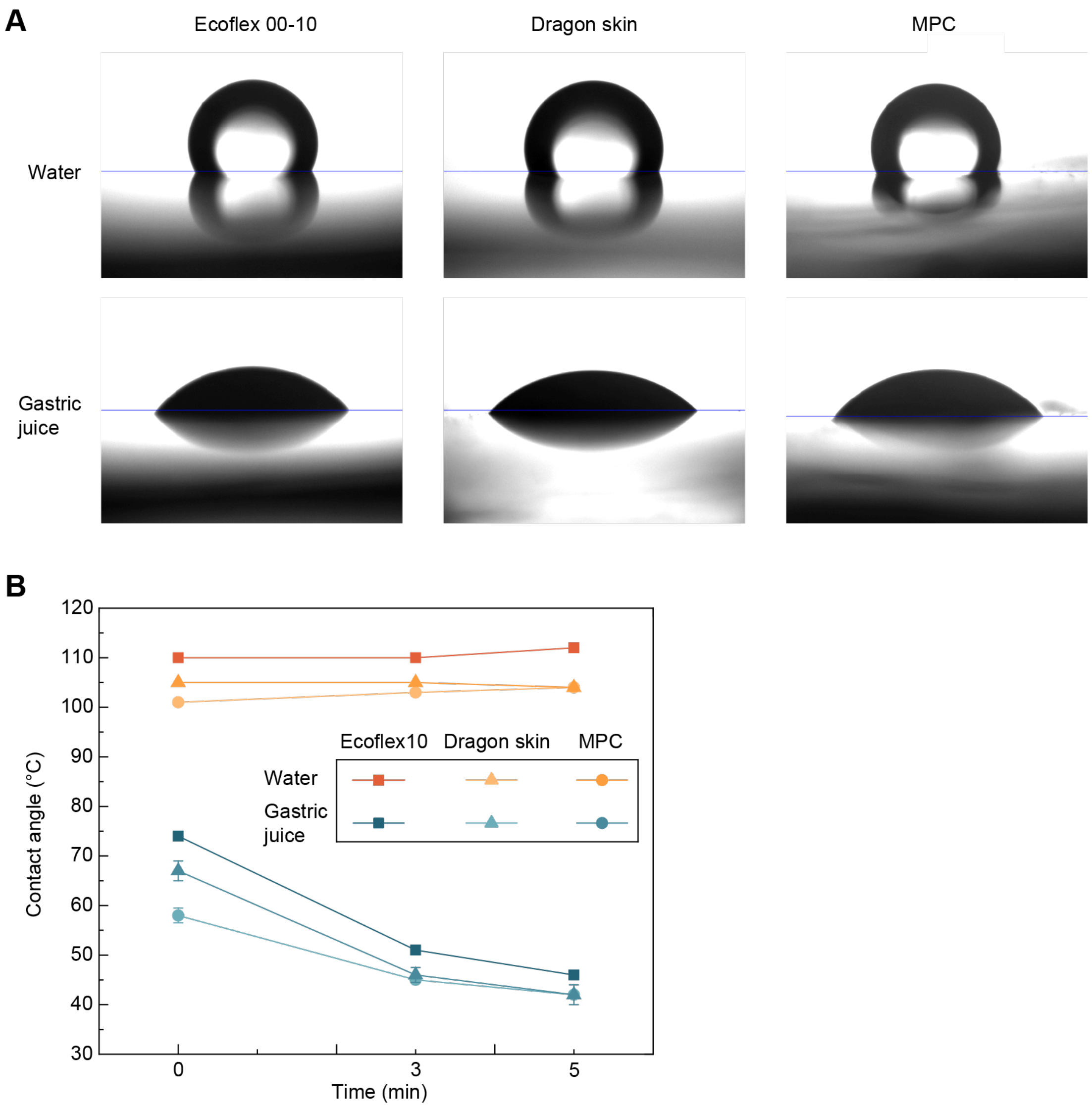

Figure S16: **Measurement of contact angle.** (**A**) Images and (**B**) comparison of contact angle between different materials and liquids.

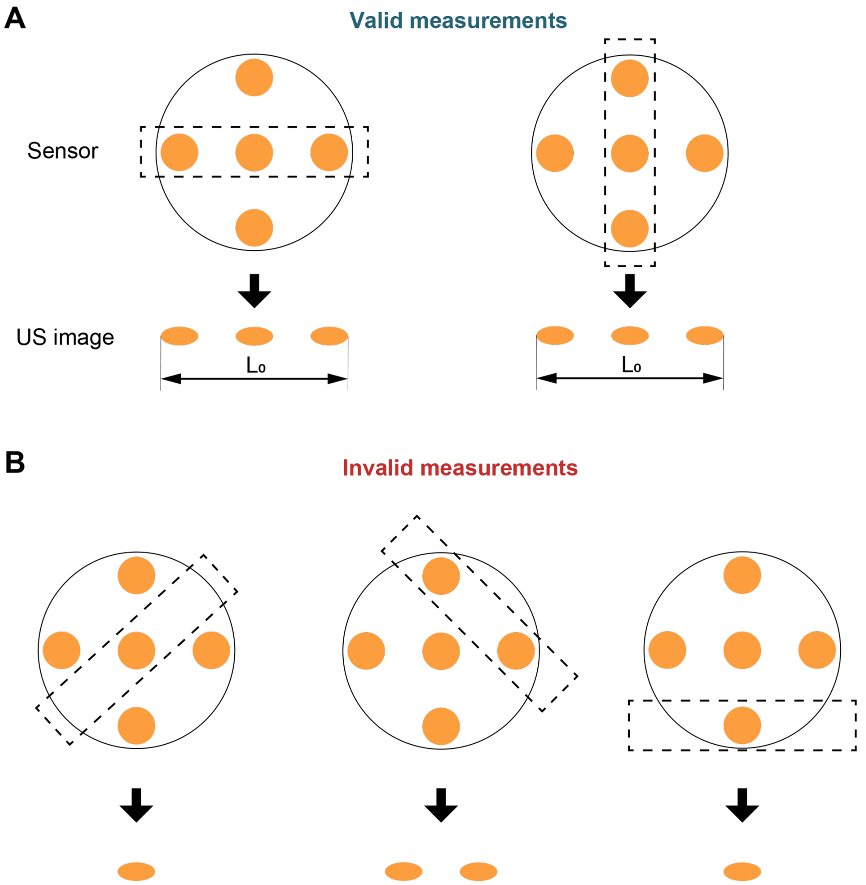

Figure S17: **Schematic illustration of the ultrasound measurement strategy for the hydrogel-based sensors.** (**A**) Valid measurements are indicated by the appearance of three high–ultrasound-contrast spots at equal distances. (**B**) Fewer than three visible spots indicates an invalid measurement.

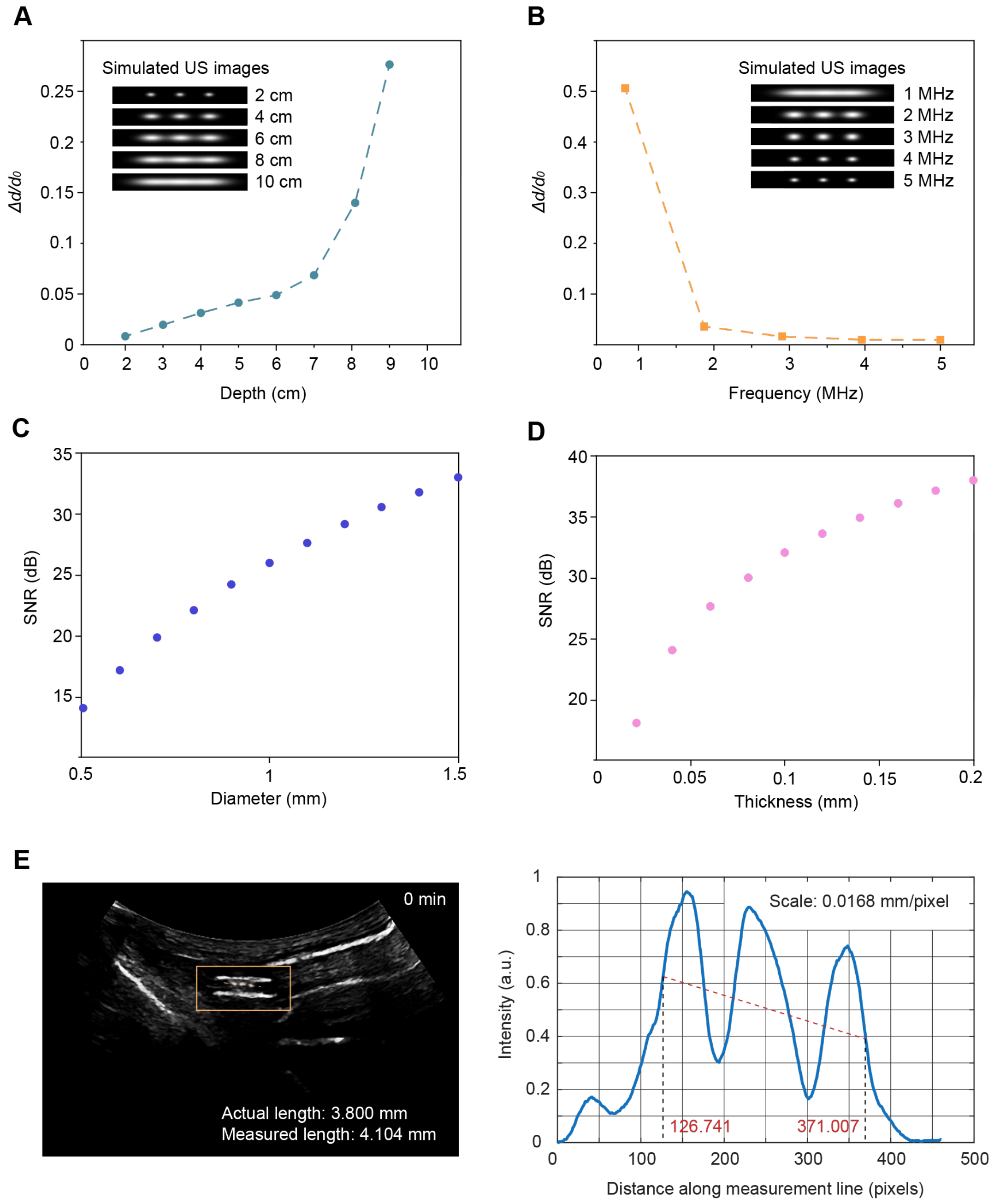

Figure S18: **Quantitative evaluation of ultrasound detection resolution.** (**A**) Numerical simulation results for the measurement accuracy at different focal depth and (**B**) ultrasound frequencies. $\Delta d/d_0$ is the deviation of measured length divided by the actual length. (**C**) Numerical simulation results for the effects of diameter and (**D**) thickness of Zn disks on the signal-to-noise (SNR) at 2 cm depth and a frequency of 5 MHz. (**E**) Examples of length measurements by MATLAB coding using the ultrasound image from SeroTab at 0 min in a rabbit model.

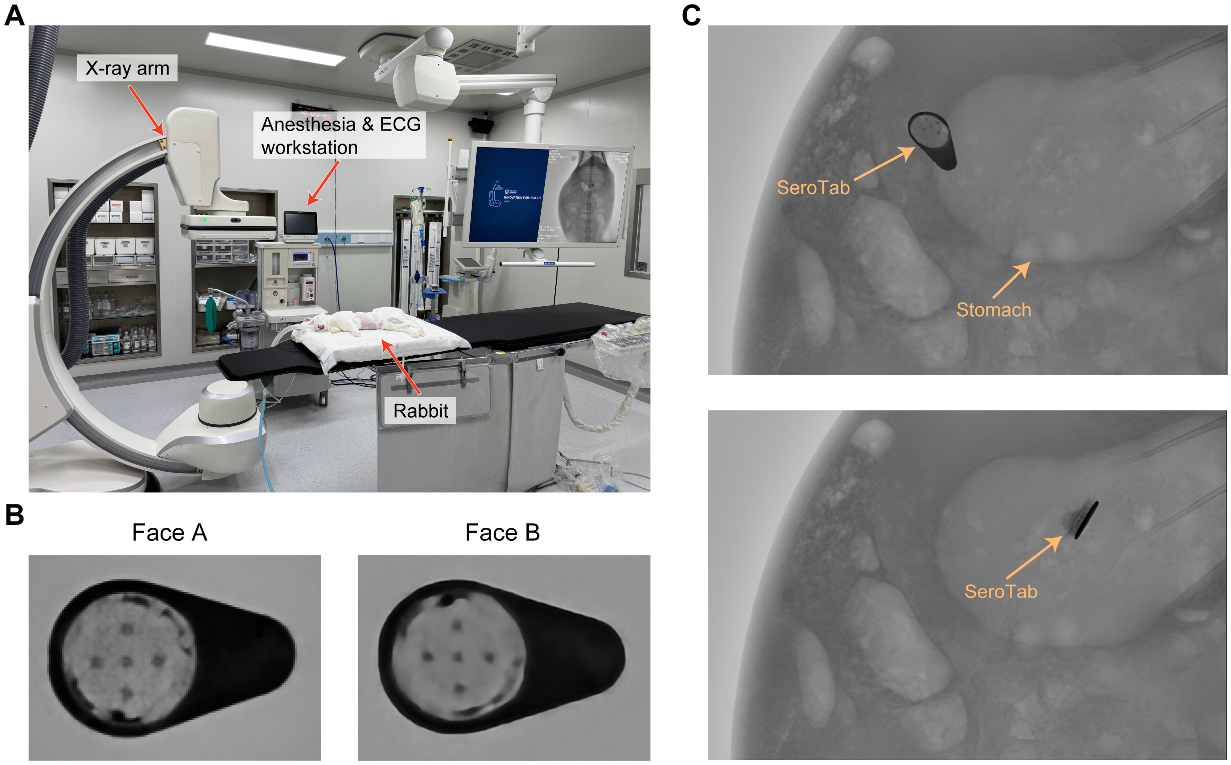

Figure S19: **X-ray imaging of SeroTab in a live rabbit stomach.** (**A**) Experimental setup. (**B**) X-ray images of the SeroTab from its two faces. (**C**) X-ray confirmation of SeroTab's position and orientation in the stomach.

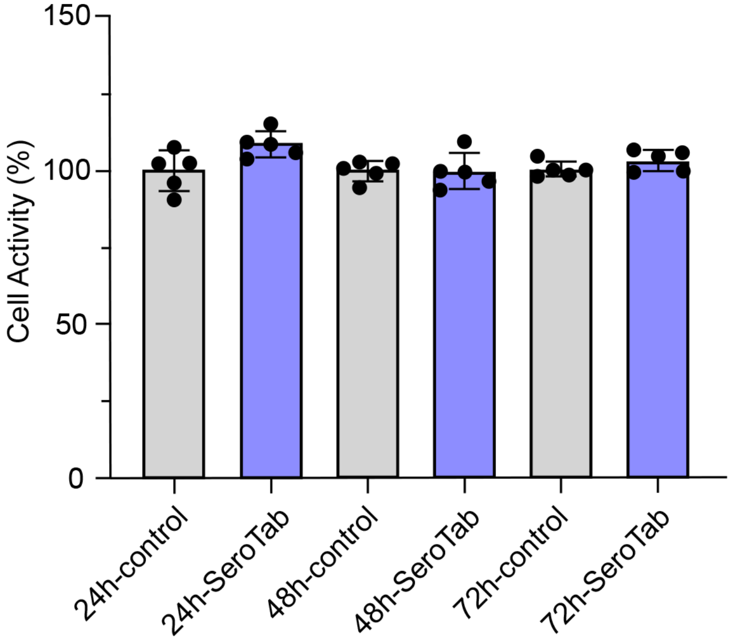

Figure S20: **Cytotoxicity evaluation of HT29 cells after 24, 48, and 72 hours of incubation with extracts from SeroTab.**

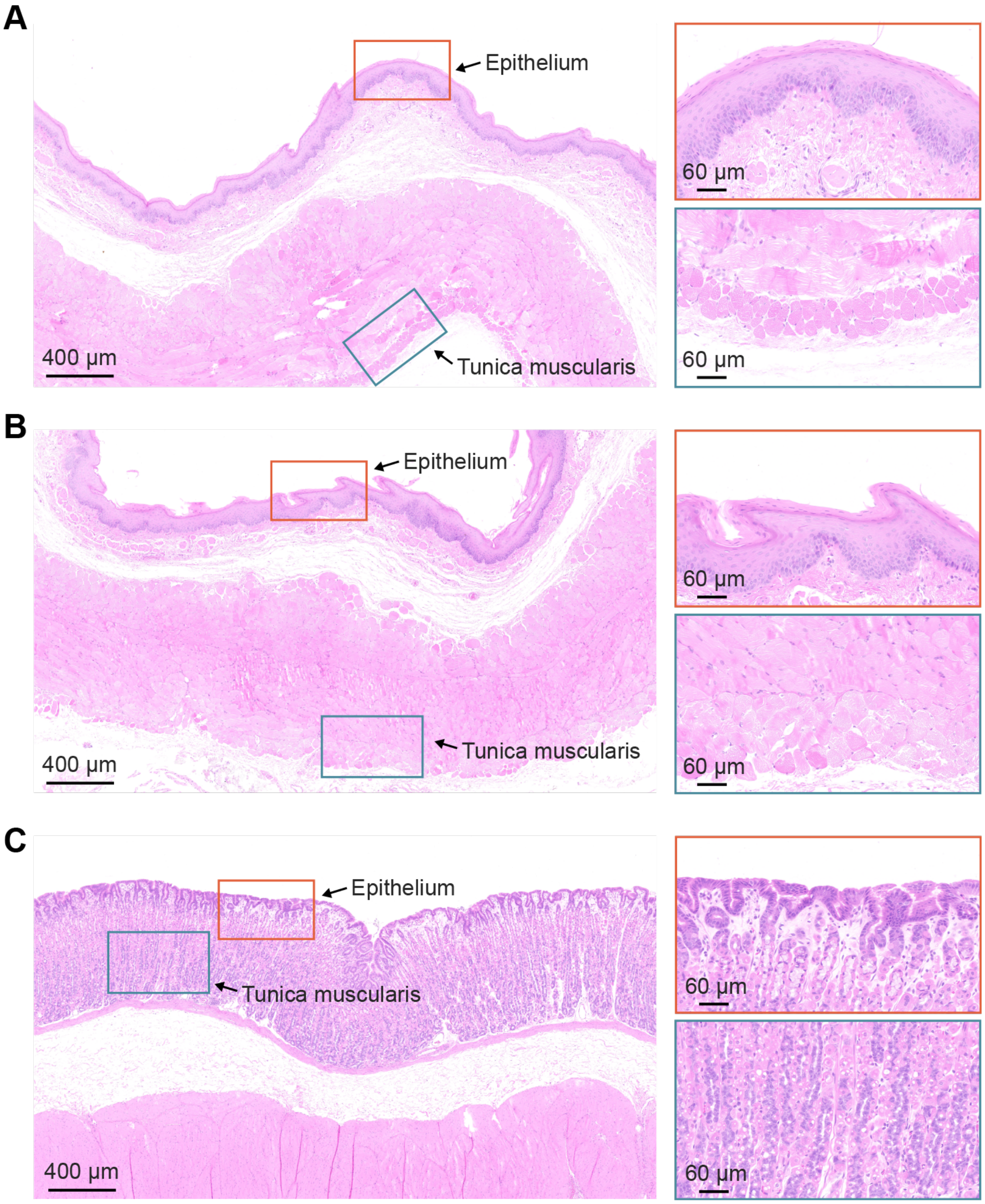

Figure S21: **Hematoxylin and eosin staining of cross sections of the esophagus and stomach after operation to assess biocompatibility.** (**A**) Control group of esophagus sample. (**B**) Experimental group of esophagus sample. (**C**) Control group of stomach sample.

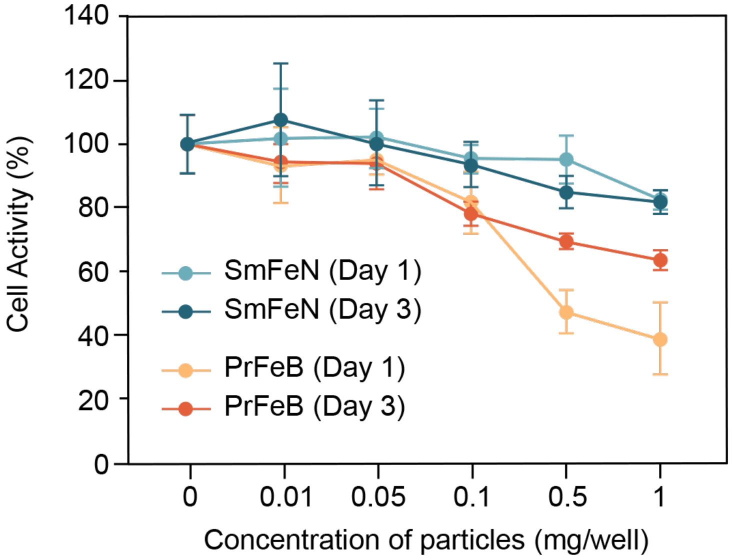

Figure S22: **Cytocompatibility of PrFeB and SmFeN toward NHDF cells.**

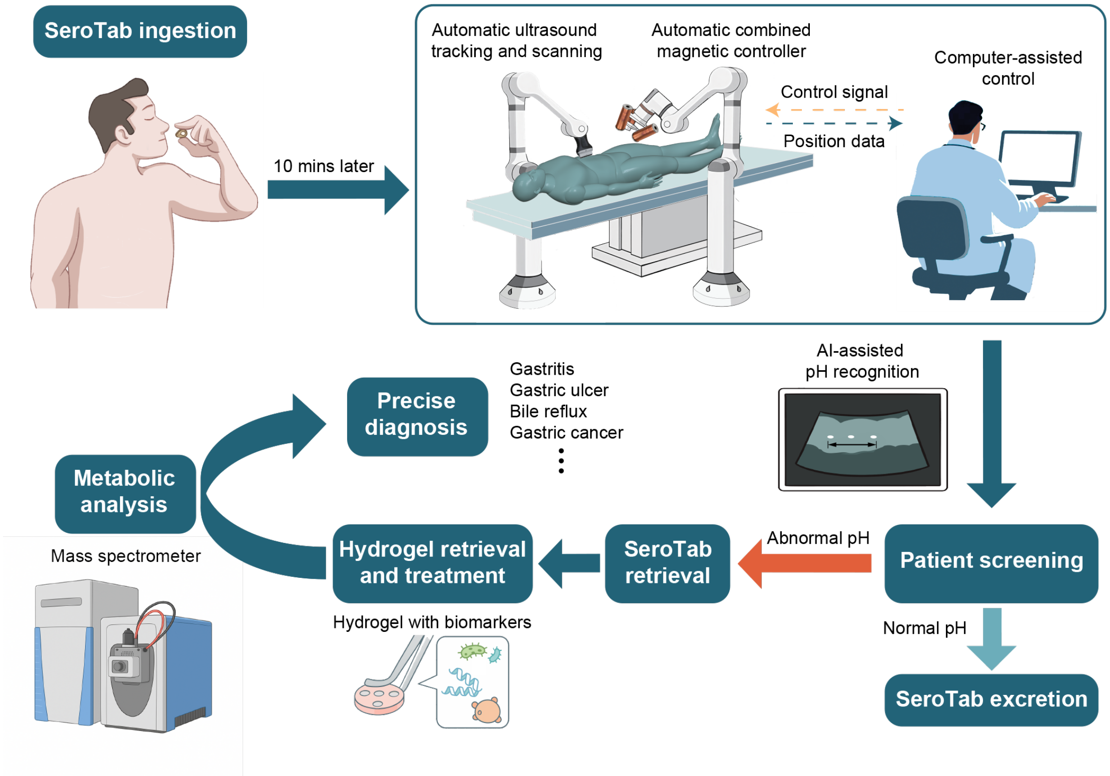

Figure S23: **Illustration of the envisioned clinical workflow for SeroTab.** Illustrations created by the authors using Adobe Photoshop and Adobe Illustrator.

Caption for Movie S1. Penguin-inspired motion pattern: posture, sliding, and orientation. Phase 1: SeroTab's body bends in response to an approaching magnet. Phase 2: SeroTab is magnetically pulled along a surface by an external moving magnet. Phase 3: SeroTab's orientation aligns with the rotation of the external magnet.

Caption for Movie S2. Penguin-inspired locomotion overcoming obstacles. Phase 1: SeroTab slides smoothly over small surface irregularities, maintaining stable motion. Phase 2: SeroTab adapts its posture to overcome larger obstacles.

Caption for Movie S3. SeroTab's motion path control on tissue surfaces *ex vivo*. SeroTab follows a pre-defined path spelling "RuG" on porcine tissue (small intestine) under external magnetic actuation.

Caption for Movie S4. SeroTab's magnetic navigation in tubular organ model *ex vivo*. SeroTab is guided through a porcine trachea via external magnetic actuation, with its motion monitored in real time using an endoscope.

Caption for Movie S5. Demonstration of locomotion and liquid sampling in porcine stomach *ex vivo*. SeroTab is magnetically guided to a target site containing gastric juice in an open porcine stomach. Upon arrival, an external radiofrequency (RF) heater is applied to activate the sampling mechanism. After fluid collection, the device is re-navigated to a second target location.

Caption for Movie S6. Demonstration of liquid sampling procedure. SeroTab is positioned with its inlet immersed in a luminous liquid. An external RF heater placed 10 mm behind the device activates the internal SMP actuator for fluid intake.

Caption for Movie S7. Swelling behavior of the hydrogel-based pH sensor in simulated gastric juice. The pH-sensitive hydrogel, embedded with Zn disks, begins to swell upon contact with simulated gastric juice (pH = 3) inside the sampling chamber of SeroTab.

Caption for Movie S8. Demonstration of SeroTab's motion in live rabbit stomach *in vivo*. Phase 1: SeroTab performs sliding motion toward the target site under magnetic guidance. Phase 2: A flipping motion enables it to overcome anatomical obstacles. Phase 3: SeroTab reorients to align its inlet with the gastric juice. Phase 4: The external magnet is removed, allowing SeroTab to be released into the gastric fluid for sampling.

Caption for Movie S9. Ultrasound imaging for SeroTab localization and observation in live rabbit stomach *in vivo*. Phase 1: Ultrasound imaging captures SeroTab flipping over within the stomach. Phase 2: SeroTab swings under the control of an external magnet, as visualized via ultrasound. Phase 3: SeroTab adjusts its posture to enhance ultrasound visibility.

Caption for Movie S10. Ultrasound imaging for pH sensor detection in live rabbit stomach *in vivo*. Phase 1: Ultrasound imaging reveals the long-axis cross section and the position of Zn disks embedded within the hydrogel pH sensor. Phase 2: Ultrasound imaging reveals the short-axis cross section and the position of Zn disks within the hydrogel pH sensor.

Caption for Movie S11. Comparative study of Tesla valve-enabled leakage prevention under impact. A 500 g weight is released onto the devices to mimic accidental mechanical shocks. The device incorporating a Tesla valve shows improved resistance to leakage compared with the straight-channel configuration.

REFERENCES

1. A. F. Peery, C. C. Murphy, C. Anderson, E. T. Jensen, S. Deutsch-Link, M. D. Egberg, J. L. Lund, D. Subramaniam, E. S. Dellon, A. D. Sperber, O. S. Palsson, Burden and cost of gastrointestinal, liver, and pancreatic diseases in the United States: Update 2024. *Gastroenterology* **168**, 1000–1024 (2025).

2. Y. Wang, Y. Huang, R. Chase, T. Li, D. Ramai, S. Li, X. Huang, S. Antwi, A. Keaveny, M. Pang, Global burden of digestive diseases: A systematic analysis of the global burden of diseases study, 1990 to 2019. *Gastroenterology* **165**, 773–783.e15 (2023).

3. W. Thompson, K. Heaton, G. Smyth, C. Smyth, Irritable bowel syndrome in general practice: Prevalence, characteristics, and referral. *Gut* **46**, 78–82 (2000).

4. B. Starfield, L. Shi, J. Macinko, Contribution of primary care to health systems and health. *Milbank Q.* **83**, 457–502 (2005).

5. C. B. Forrest, Primary care gatekeeping and referrals: Effective filter or failed experiment? *BMJ* **326**, 692–695 (2003).

6. D. Kringos, W. Boerma, Y. Bourgueil, T. Cartier, T. Dedeu, T. Hasvold, A. Hutchinson, M. Lember, M. Oleszczyk, D. Pavlic, I. Svab, The strength of primary care in Europe: An international comparative study. *Br. J. Gen. Pract.* **63**, e742–e750 (2013).

7. World Health Organization, *Medical Devices: Managing the Mismatch: An Outcome of the Priority Medical Devices Project* (World Health Organization, 2010).

8. M. Schubert, D. Peura, Control of gastric acid secretion in health and disease. *Gastroenterology* **134**, 1842–1860 (2008).

9. J. D. Gardner, Increased gastric acid secretion as a possible cause of GERD. *Nat. Rev. Gastroenterol. Hepatol.* **7**, 125–126 (2010).

10. W. De Vos, H. Tilg, M. Van Hul, P. Cani, Gut microbiome and health: Mechanistic insights. *Gut* **71**, 1020–1032 (2022).

11. Q. Ci, Y. Wang, B. Wu, E. Coy, J. Li, D. Jiang, P. Zhang, G. Wang, Fe-doped carbon dots as NIR-II fluorescence probe for in vivo gastric imaging and pH detection. *Adv. Sci.* **10**, e2206271 (2023).

12. W. Huang, R. Chen, Y. Peng, F. Duan, Y. Huang, W. Guo, X. Chen, L. Nie, In vivo quantitative photoacoustic diagnosis of gastric and intestinal dysfunctions with a broad pH-responsive sensor. *ACS Nano* **13**, 9561–9570 (2019).

13. T. Ghosh, D. Lewis, A. Axon, S. Everett, Methods of measuring gastric acid secretion. *Aliment. Pharmacol. Ther.* **33**, 768–781 (2011).

14. M. Chiara, M. Florenzo, R. Michele, S. Serena, F. Marilisa, C. Pellegrino, F. Lorella, N. Antonio, M. Tiziana, L. Gioacchino, L. Gian, Non-invasive method for the assessment of gastric acid secretion. *Acta Biomed. Aten. Parm.* **89**, 53 (2018).

15. W. Steinberg, F. Mina, P. Pick, G. Frey, Heidelberg capsule I: In vitro evaluation of a new instrument for measuring intragastric pH. *J. Pharm. Sci.* **54**, 772–776 (1965).

16. B. Jacobson, R. Mackay, A pH-endoradiosonde. *Lancet* **269**, 1224 (1957).

17. X. Zhang, G. Chen, H. Zhang, L. Shang, Y. Zhao, Bioinspired oral delivery devices. *Nat. Rev. Bioeng.* **1**, 208–225 (2023).

18. J. Min, H. Ahn, H. Lukas, X. Ma, R. Bhansali, S. Sunwoo, C. Wang, Y. Xu, D. Yao, G. Kim, Z. Li, T. K. Hsiai, A. Emami, H.-T. Jung, W. Gao, Continuous biochemical profiling of the gastrointestinal tract using an integrated smart capsule. *Nat. Electron.* **8**, 844–855 (2025).

19. M. Rehan, I. Al-Bahadly, D. Thomas, W. Young, L. Cheng, E. Avci, Smart capsules for sensing and sampling the gut: Status, challenges and prospects. *Gut* **73**, 186–202 (2024).

20. W. Hu, G. Lum, M. Mastrangeli, M. Sitti, Small-scale soft-bodied robot with multimodal locomotion. *Nature* **554**, 81–85 (2018).

21. J. Cui, T. Huang, Z. Luo, P. Testa, H. Gu, X. Chen, B. Nelson, L. Heyderman, Nanomagnetic encoding of shape-morphing micromachines. *Nature* **575**, 164–168 (2019).

22. X. Zhao, Y. Kim, Magnetic soft materials and robots. *Chem. Rev.* **122**, 5317–5364 (2022).

23. S. Tottori, L. Zhang, F. Qiu, K. Krawczyk, A. Franco-Obregón, B. Nelson, Magnetic helical micromachines: Fabrication, controlled swimming, and cargo transport. *Adv. Mater.* **24**, 811–816 (2012).

24. T. Xu, J. Zhang, M. Salehizadeh, O. Onaizah, E. Diller, Millimeter-scale flexible robots with programmable three-dimensional magnetization and motions. *Sci. Robot.* **4**, eaav4494 (2019).

25. H. Xie, M. Sun, X. Fan, Z. Lin, W. Chen, L. Wang, L. Dong, Q. He, Reconfigurable magnetic microrobot swarm: Multimode transformation, locomotion, and manipulation. *Sci. Robot.* **4**, eaav8006 (2019).

26. C. Wang, A. Mzyk, R. Schirhagl, S. Misra, V. Venkiteswaran, Biocompatible film-coating of magnetic soft robots for mucoadhesive locomotion. *Adv. Mater. Technol.* **8**, 2201813 (2023).

27. T. Wang, Y. Wu, E. Yildiz, S. Kanyas, M. Sitti, Clinical translation of wireless soft robotic medical devices. *Nat. Rev. Bioeng.* **2**, 470–485 (2024).

28. S. Srinivasan, A. Alshareef, A. Hwang, Z. Kang, J. Kuosmanen, K. Ishida, J. Jenkins, S. Liu, W. Madani, J. Lennerz, RoboCap: Robotic mucus-clearing capsule for enhanced drug delivery in the gastrointestinal tract. *Sci. Robot.* **7**, eabp9066 (2022).

29. R. Mundaca-Uribe, N. Askarinam, R. Fang, L. Zhang, J. Wang, Towards multifunctional robotic pills. *Nat. Biomed. Eng.* **8**, 1334–1346 (2024).

30. X. Dong, B. Xiao, H. Vu, H. Lin, M. Sitti, Millimeter-scale soft capsules for sampling liquids in fluid-filled confined spaces. *Sci. Adv.* **10**, eadp2758 (2024).

31. N. Mandsberg, G. Moro, M. Ghavami, S. Andersen, E. de Visser, M. Bertelsen, M. Mortensen, T. Licht, A. Boisen, Ingestible device for gastric fluid sampling. *Adv. Mater. Technol.* **9**, 2400434 (2024).

32. A. Abramson, M. Frederiksen, A. Vegge, B. Jensen, M. Poulsen, B. Mouridsen, M. Jespersen, R. Kirk, J. Windum, F. Hubálek, J. Water, Oral delivery of systemic monoclonal antibodies, peptides and small molecules using gastric auto-injectors. *Nat. Biotechnol.* **40**, 103–109 (2022).

33. D. Son, H. Gilbert, M. Sitti, Magnetically actuated soft capsule endoscope for fine-needle biopsy. *Soft Robot.* **7**, 10–21 (2020).

34. Y. Sun, W. Zhang, J. Gu, L. Xia, Y. Cao, X. Zhu, H. Wen, S. Ouyang, R. Liu, J. Li, Z. Jiang, Magnetically driven capsules with multimodal response and multifunctionality for biomedical applications. *Nat. Commun.* **15**, 1839 (2024).

35. R. Del-Rio-Ruiz, D. da Silva, H. Suresh, H. Creasey, C. Asci, D. dos Santos, A. Sharma, G. Widmer, S. Sonkusale, Soft autonomous ingestible device for sampling the small-intestinal microbiome. *Device* **2**, 100406 (2024).

36. Y. Lai, T. Lee, D. Sieben, L. Gauthier, J. Nam, E. Diller, Hybrid hydrogel-magnet actuated capsule for automatic gut microbiome sampling. *IEEE Trans. Biomed. Eng.* **71**, 2911–2922 (2024).

37. S. Park, M. Hoang, J. Kim, S. Park, Multiple sampling capsule robot for studying gut microbiome. *Adv. Intell. Syst.* **7**, 2300625 (2025).

38. D. Li, J. Zhou, Z. Zhao, X. Huang, H. Li, Q. Qu, C. Zhou, K. Yao, Y. Liu, M. Wu, J. Su, Battery-free, wireless, and electricity-driven soft swimmer for water quality and virus monitoring. *Sci. Adv.* **10**, eadk6301 (2024).

39. S. Madhvapathy, M. Bury, L. Wang, J. Ciatti, R. Avila, Y. Huang, A. Sharma, J. Rogers, Miniaturized implantable temperature sensors for the long-term monitoring of chronic intestinal inflammation. *Nat. Biomed. Eng.* **8**, 1040–1052 (2024).

40. J. Rivnay, P. Sheehan, O. Veiseh, Are implantable, living pharmacies within reach? *Science* **386**, 271–273 (2024).

41. J. Liu, N. Liu, Y. Xu, M. Wu, H. Zhang, Y. Wang, Y. Yan, A. Hill, R. Song, Z. Xu, M. Park, Y. Wu, J. L Ciatti, J. Gu, H. Luan, Y. Zhang, T. Yang, H.-Y. Ahn, S. Li, W. Z. Ray, C. K. Franz, M. R. Macewan, Y. Huang, C. W. Hammill, H. Wang, J. A. Rogers, Bioresorbable shape-adaptive structures for ultrasonic monitoring of deep-tissue homeostasis. *Science* **383**, 1096–1103 (2024).

42. H. Tang, Y. Yang, Z. Liu, W. Li, Y. Zhang, Y. Huang, T. Kang, Y. Yu, N. Li, Y. Tian, X. Liu, Injectable ultrasonic sensor for wireless monitoring of intracranial signals. *Nature* **630**, 84–90 (2024).

43. Q. Wang, S. Yang, L. Zhang, Untethered micro/nanorobots for remote sensing: Toward intelligent platform. *Nanomicro Lett.* **16**, 40 (2024).

44. C. Wang, Y. Wu, X. Dong, M. Armacki, M. Sitti, In situ sensing physiological properties of biological tissues using wireless miniature soft robots. *Sci. Adv.* **9**, eadg3988 (2023).

45. J. Han, X. Dong, Z. Yin, S. Zhang, M. Li, Z. Zheng, M. Ugurlu, W. Jiang, H. Liu, M. Sitti, Actuation-enhanced multifunctional sensing and information recognition by magnetic artificial cilia arrays. *Proc. Natl. Acad. Sci. U.S.A.* **120**, e2308301120 (2023).

46. Z. Wang, Y. Wu, B. Zhu, Q. Chen, L. Wang, Y. Zhao, D. Sun, J. Zheng, D. Wu, A magnetic soft robot with multimodal sensing capability by multimaterial direct ink writing. *Addit. Manuf.* **61**, 103320 (2023).

47. S. Li, D. Lu, S. Li, J. Liu, Y. Xu, Y. Yan, J. Rodriguez, H. Bai, R. Avila, S. Kang, X. Ni, Bioresorbable, wireless, passive sensors for continuous pH measurements and early detection of gastric leakage. *Sci. Adv.* **10**, eadj0268 (2024).

48. C. Wang, X. Chen, L. Wang, M. Makihata, H. Liu, T. Zhou, X. Zhao, Bioadhesive ultrasound for long-term continuous imaging of diverse organs. *Science* **377**, 517–523 (2022).

49. H. Hu, H. Huang, M. Li, X. Gao, L. Yin, R. Qi, R. Wu, X. Chen, Y. Ma, K. Shi, C. Li, A wearable cardiac ultrasound imager. *Nature* **613**, 667–675 (2023).

50. M. Wehner, R. Truby, D. Fitzgerald, B. Mosadegh, G. Whitesides, J. Lewis, R. Wood, An integrated design and fabrication strategy for entirely soft, autonomous robots. *Nature* **536**, 451–455 (2016).

51. L. Masjosthusmann, M. Richter, P. Makushko, D. Makarov, S. Misra, Miniaturized variable stiffness gripper locally actuated by magnetic fields. *Adv. Intell. Syst.* **6**, 2400037 (2024).

52. R. Soon, Z. Yin, M. Dogan, N. Dogan, M. Tiryaki, A. Karacakol, A. Aydin, P. Esmaeili-Dokht, M. Sitti, Pangolin-inspired untethered magnetic robot for on-demand biomedical heating applications. *Nat. Commun.* **14**, 3320 (2023).

53. S. Beg, K. Ragunath, A. Wyman, M. Banks, N. Trudgill, M. Pritchard, S. Riley, J. Anderson, H. Griffiths, P. Bhandari, P. Kaye, Quality standards in upper gastrointestinal endoscopy: A position statement of the British Society of Gastroenterology (BSG) and Association of Upper Gastrointestinal Surgeons of Great Britain and Ireland (AUGIS). *Gut* **66**, 1886–1899 (2017).

54. M. Rezapour, C. Amadi, L. Gerson, Retention associated with video capsule endoscopy: Systematic review and meta-analysis. *Gastrointest. Endosc.* **85**, 1157–1168.e2 (2017).

55. C. Thorndal, O. Selnes, I. Lei, A. Koulaouzidis, A systematic review of capsule aspiration in capsule endoscopy. *Ann. Transl. Med.* **12**, 12 (2023).

56. F. Taktak, Rapid deswelling of PDMAEMA hydrogel in response to pH and temperature changes and its application in controlled drug delivery. *AKU J. Sci. Eng.* **16**, 68–75 (2016).

57. L. Kong, X. Lu, X. Bian, W. Zhang, C. Wang, Constructing carbon-coated Fe_3O_4 microspheres as antiacid and magnetic support for palladium nanoparticles for catalytic applications. *ACS Appl. Mater. Interfaces* **3**, 35–42 (2011).

58. L. Yin, H. Cheng, S. Mao, R. Haasch, Y. Liu, X. Xie, S. Hwang, H. Jain, S. Kang, Y. Su, R. Li, Dissolvable metals for transient electronics. *Adv. Funct. Mater.* **24**, 645–658 (2014).

59. J. Chen, A. Alexiev, A. Sergnese, N. Fabian, A. Pettinari, Y. Cai, V. Perepelook, K. Schmidt, A. Hayward, A. Guevara, B. Laidlaw, An ingestible capsule for luminance-based diagnosis of mesenteric ischemia. *Sci. Robot.* **10**, eadx1367 (2025).

60. N. Deirram, C. Zhang, S. Kermaniyan, A. Johnston, G. Such, pH-responsive polymer nanoparticles for drug delivery. *Macromol. Rapid Commun.* **40**, 1800917 (2019).

61. W. Lee, H. Wan, Z. Cheng, W. Chan, T. Lam, K. Lai, J. Wang, Z. Cai, C. Wong, Impact of PFOS exposure on murine fetal hematopoietic stem cells, associated with intrauterine metabolic perturbation. *Environ. Sci. Technol.* **59**, 5496–5509 (2025).

62. Z. He, H. Zhang, Y. Song, Z. Yang, Z. Cai, Exposure to ambient fine particulate matter impedes the function of spleen in the mouse metabolism of high-fat diet. *J. Hazard. Mater.* **423**, 127129 (2022).

63. Y. Song, Y. Zhang, L. Zhu, Y. Chen, Y. Chen, Z. Zhu, J. Feng, Z. Qi, J. Yu, Z. Yang, Z. Cai, Phosphocholine-induced energy source shift alleviates mitochondrial dysfunction in lung cells caused by geospecific PM2.5 components. *Proc. Natl. Acad. Sci. U.S.A.* **121**, e2317574121 (2024).

64. B. Figliuzzi, C. Buie, Rise in optimized capillary channels. *J. Fluid Mech.* **731**, 142–161 (2013).

65. N. Ichikawa, K. Hosokawa, R. Maeda, Interface motion of capillary-driven flow in rectangular microchannel. *J. Colloid Interface Sci.* **280**, 155–164 (2004).

66. J. Cai, T. Jin, J. Kou, S. Zou, J. Xiao, Q. Meng, Lucas-Washburn equation-based modeling of capillary-driven flow in porous systems. *Langmuir* **37**, 1623–1636 (2021).

67. P. Hu, P. Wang, L. Liu, X. Ruan, L. Zhang, Z. Xu, Numerical investigation of Tesla valves with a variable angle. *Phys. Fluids* **34**, 033603 (2022).

68. Q. Nguyen, J. Abouezzi, L. Ristroph, Early turbulence and pulsatile flows enhance diodicity of Tesla's macrofluidic valve. *Nat. Commun.* **12**, 2884 (2021).
